# Quantification and stability assessment of urinary phenolic and acidic biomarkers of non-persistent chemicals using the SPE-GC/MS/MS method

**DOI:** 10.1007/s00216-023-04633-7

**Published:** 2023-03-18

**Authors:** Anna Klimowska, Evelien Wynendaele, Bartosz Wielgomas

**Affiliations:** 1 Department of Toxicology, Faculty of Pharmacy, Medical University of Gdańsk, 107 Hallera Street, 80-416 Gdańsk, Poland; 2 DruQuaR Laboratory, Faculty of Pharmaceutical Sciences, Ottergemse Steenweg 460, 9000 Ghent, Belgium

**Keywords:** Phenolic biomarkers, SPE, Short- and long-term storage stability, Urine, Validation

## Abstract

Nowadays, people are exposed to numerous man-made chemicals, many of which are ubiquitously present in our daily lives, and some of which can be hazardous to human health. Human biomonitoring plays an important role in exposure assessment, but complex exposure evaluation requires suitable tools. Therefore, routine analytical methods are needed to determine several biomarkers simultaneously. The aim of this study was to develop an analytical method for quantification and stability testing of 26 phenolic and acidic biomarkers of selected environmental pollutants (e.g., bisphenols, parabens, pesticide metabolites) in human urine. For this purpose, a solid-phase extraction coupled with gas chromatography and tandem mass spectrometry (SPE-GC/MS/MS) method was developed and validated. After enzymatic hydrolysis, urine samples were extracted using Bond Elut Plexa sorbent, and prior to GC, the analytes were derivatized with *N*-trimethylsilyl-*N*-methyl trifluoroacetamide (MSTFA). Matrix-matched calibration curves were linear in the range of 0.1–1000 ng mL^−1^ with R > 0.985. Satisfactory accuracy (78–118%), precision (< 17%), and limits of quantification (0.1–0.5 ng mL^−1^) were obtained for 22 biomarkers. The stability of the biomarkers in urine was assayed under different temperature and time conditions that included freezing and thawing cycles. All tested biomarkers were stable at room temperature for 24 h, at 4 °C for 7 days, and at −20 °C for 18 months. The total concentration of 1-naphthol decreased by 25% after the first freeze–thaw cycle. The method was successfully used for the quantification of target biomarkers in 38 urine samples.

**Supplementary Information:**

The online version contains supplementary material available at 10.1007/s00216-023-04633-7.

## Introduction

The continuous technological and scientific development contributes to introducing new chemicals into many consumer products, such as food, medicines, or personal care products. As a result, people are constantly exposed to a mixture of numerous chemicals, and there is an urgent need for performing human health risk assessment. The major question is how much of these substances has entered the human body and what negative health effects may result from it. One of the best tools for estimating internal exposure is human biomonitoring (HBM), which is based on the measurement of the concentration of a chemical substance or its metabolite in human biological fluids. For example, urinary concentration of certain metabolites can be utilized to back-calculate the absorbed dose, which in turn can be compared to health-based exposure limits, such as tolerable daily intake (TDI). Since environmental pollutants (parent compounds) or their metabolites are present in biological samples in trace amounts, multi-analyte, reliable, and robust analytical methods are constantly needed. However, published methods are mostly limited to the determination of substances from a defined chemical group, such as phthalate metabolites or phenolic compounds. Due to their similar physicochemical properties, it is much easier to find the right conditions for sample preparation as well as instrumental analysis. However, this approach often requires collecting a larger volume of biological material, since a single sample is examined using more than one analytical method. Alternatively, a method may use a single sample preparation procedure, but require multiple injections [1]. Therefore, multi-analyte gas chromatography/mass spectrometry (GC/MS) and liquid chromatography/mass spectrometry (LC/MS) methods that are capable of quantifying several biomarkers with sufficient sensitivity in a single chromatographic run are currently required. There are many benefits to this approach, such as reduced costs, increased time savings, and a more eco-friendly process by limiting the use of harmful analytical chemicals.

A recent HBM4EU initiative prioritized 17 groups of chemicals for which biomarkers of exposure have been measured in biological samples collected from European citizens [2]. Based on this list, and the data on the prevalence of exposure, toxicity, and toxicokinetics of the parent compounds, 26 biomarkers of exposure of similar physicochemical properties were selected for this study. Since the analysis of phenols and carboxylic acids requires a derivatization step when using gas chromatography (GC), liquid chromatography coupled with tandem mass spectrometry (LC/MS/MS) is usually preferred [3–8]. Prior to instrumental analysis, biomarkers are isolated from the urine sample using classic solid-phase extraction (SPE) [3, 4, 9] or online SPE coupled to an LC system [5, 6, 8]. However, the GC/MS technique is still less expensive and easier to maintain and operate than LC/MS, hence some GC/MS methods have been published recently [9–11]. In the study by Pirard et al. [9], bisphenol A (BPA), triclosan (TCS), and 4-nonylphenol were isolated from urine using OASIS HLB sorbent and then recovered with a mixture of methanol and dichloromethane (50:50, *v*:*v*). Azzouz et al. [10] extracted 13 phenols from urine by liquid–liquid extraction (LLE) with ethyl acetate, and the extract was subsequently cleaned by an automated SPE system. Lu et al. [11] isolated 20 phenols from urine using a mixture of methyl *tert*-butyl ether and *n*-hexane (1:3, *v:v*), then cleaning the extract by K_2_CO_3_ treated silica-based SPE. Prior to GC analysis, phenols were most frequently derivatized with silylating agents [10–12].

Taking into account that persistence in the environment and the ability to accumulate in living organisms is an undesirable feature of many synthetic chemicals, most of the currently used substances have a relatively short biological half-life (hours or days). After entering the human body, they are rapidly metabolized and excreted in the urine. Representatives of this group are phenolic compounds (e.g., bisphenols, parabens, oxybenzone, triclosan) and some pesticides (e.g., synthetic pyrethroids, neonicotinoids). To assess the exposure level to these compounds, the total biomarker concentrations are measured in urine samples. In HBM studies, individual samples are usually collected from study participants over a longer period of time; hence the samples may be stored frozen pending analysis for several months or years. Only two studies report the long-term stability of phenolic compounds in urine samples over 6 months or 1 year when stored at −70 °C or −80 °C, respectively [12, 13]. In addition, freeze–thaw cycles may also affect the stability of phenolic compounds; but there are still scarce data on this issue. Regarding the analytes presented in this study, we found such data only for 1-naphthol (1-NP) and 2-naphthol (2-NP) [12].

This study aimed to (i) develop an SPE-GC/MS/MS method for the quantitative determination of several biomarkers of non-persistent environmental pollutants, and (ii) investigate the effects of different storage and thawing conditions on the stability of biomarkers in urine samples.

## Materials and methods

### Reagents and materials

Analytical and internal standards including 2,4-dichlorophenol (2,4-DCP), 2-naphthol (2-NP), 3-phenoxybenzoic acid (3-PBA), 6-chloronicotinic acid (6-CNA), benzophenone 1 (BP-1), benzophenone 3 (BP-3), bisphenol A (BPA), bisphenol B (BPB), bisphenol BP (BPBP), bisphenol C (BPC), bisphenol E (BPE), bisphenol F (BPF), bisphenol G (BPG), bisphenol S (BPS), butylparaben (BuP), ethylparaben (EtP), isobutylparaben (iBuP), methylparaben (MeP), propylparaben (PrP), pentachlorophenol (PCP), triclosan (TCS), 2,4-dichlorophenol-3,5,6-d_3_ (2,4-DCP-d_3_), 2-phenoxybenzoic acid (2-PBA), benzophenone 3-d_5_ (BP3-d_5_, phenyl-d_5_), and bisphenol A d_16_ (BPA-d_16_) were purchased from Sigma-Aldrich (Darmstadt, Germany), while 1-naphthol (1-NP), 2,5-dichlorophenol (2,5-DCP), and 3,5,6-trichloro-2-pyridinol (TCPyr) were purchased from Riedel-de Häen (France) and 4-hydroxy-3-phenoxybenzoic acid (4OH3PBA) from Lancaster (England). The analytical standards of bisphenol AF (BPAF) and labeled analogs of 1-NP (1-NP-d_7_), 2-NP (2-NP-d_7_), BPAF (BPAF-d_4_), BPF (BPF-d_10_), BPS (BPS-d_8_), MeP (MeP-d_4_, ring-d_4_), and TCS (TCS-d_3_) were obtained from Toronto Research Chemicals (Canada). Standard solutions of butylparaben-^13^C_6_ (BuP-^13^C_6_, ring-^13^C_6_, 1 mg mL^−1^ in MeOH), propylparaben-^13^C_6_ (PrP-^13^C_6_, ring-^13^C_6_, 1 mg mL^−1^ in MeOH) and 3,5,6-trichloro-2-pyridinol-4,5,6-^13^C_3_ (TCPyr-^13^C_3_, 100 µg mL^−1^ in acetonitrile) were purchased from LGC Standards (Łomianki, Poland).

Other reagents: acetonitrile, β-glucuronidase type HP-2 from *Helix pomatia* (G7017, activity: 112,187 U mL^−1^ of β-glucuronidase and 1051 U mL^−1^ of sulfatase), and formic acid (HCOOH) were procured from Sigma-Aldrich (Darmstadt, Germany); ethyl acetate for GC/MS (EA) and methanol from Merck KGaA (Darmstadt, Germany); MSTFA from Macherey-Nagel (Dueren, Germany); and sodium acetate from POCH (Gliwice, Poland). Deionized water was obtained from a Hydrolab Basic 5 system (Hydrolab, Straszyn, Poland).

SPE columns, namely Agilent Bond Elut Plexa (30 mg, 1 mL), Supel-Select HLB (30 mg, 1 mL), and Waters OASIS HLB, were obtained from Perlan Technologies (Warsaw, Poland), Sigma-Aldrich (Darmstadt, Germany), and Waters (Warsaw, Poland), respectively.

Standard stock solutions (1 mg mL^−1^) and working solutions, a serial dilution of stock solutions, were prepared in acetonitrile. Separate working solutions were prepared for calibration and quality control assessment. An internal standard (IS) mixed solution of 1-NP-d_7_, 2-NP-d_7_, 2,4-DCP-d_3_, BP3-d_5_, BPA-d_16_, BPAF-d_4_, BPF-d_10_, BPS-d_8_, BuP-^13^C_6_, MeP-d_4_, PrP-^13^C_6_ (2.5 µg mL^−1^), and TCPyr-^13^C_3_ (1.5 µg mL^−1^) was prepared in acetonitrile. Stock solutions of BPA, BPF, and BPS glucuronides (1 mg mL^−1^) were prepared in methanol. All standard solutions were stored at −20 °C.

The mixture of acetate buffer solution (1 M, pH 5.0) and β-glucuronidase was freshly prepared prior to extraction of each batch of samples.

### Urine samples

#### Method development

Urine samples used for method development and validation were collected from six healthy volunteers (laboratory staff of the Department of Toxicology, Medical University of Gdańsk) with no occupational exposure and expected low levels of environmental exposure to the target analytes. Since exposure to compounds present in personal care products (e.g., parabens, TCS, BP-3) can be partially controlled, we checked the components of their daily personal care products. To prepare a blank urine sample, volunteers with expected low exposure to the studied compounds (absence of target chemicals in the currently used personal care products) were selected to donate urine samples. All individual samples were donated anonymously, and volunteers collected one random urine void in a 2000 mL polypropylene (PP) container. After collection, all samples were homogenized and pooled, and 2 mL aliquots were pipetted into glass screw-cap tubes and stored at −20 °C until analysis. The 2 mL aliquots were used as blank urine samples. Before analysis, they were thawed and spiked with working solutions containing all target analytes and internal standards.

#### Storage stability of biomarkers in urine

Since the glucuronides and sulfates of most of the analyzed biomarkers are not commercially available, it was decided to conduct stability studies using a urine sample donated by a volunteer with an expected high exposure to compounds present in personal care products used daily. After collection, the urine was additionally spiked with commercially available BPA, BPF, and BPS β-glucuronides at a concentration of 5 ng mL^−1^. The homogenized sample was divided into 5 mL aliquots, transferred into 20 mL PP scintillation vials, and stored at room temperature (RT) for up to 3 days, at 4 °C for up to 2 months, and −20 °C for 18 months. To assess the stability of conjugates, each urine aliquot was analyzed twice: without hydrolysis to measure the concentration of the free form and after hydrolysis to measure the total concentration of the biomarker at different time points at each temperature tested.

In addition, to assess the stability after freeze–thaw cycles, 10 mL urine aliquots were prepared and stored at −20 °C. After at least a week, they were thawed in a dark cabinet for a few hours (at RT) or in the refrigerator overnight (at 4 °C). For each condition, three replicates underwent three cycles of freezing and thawing.

No other substances, such as preservatives, were added to the containers throughout the study period. To evaluate the initial concentration of the biomarkers (day 0), the sample was analyzed by the SPE-GC/MS/MS method on the day of sampling.

### SPE procedure

Prior to SPE, the urine sample was enzymatically treated using previously established conditions [14]. Briefly, 2 mL of urine was pipetted into a glass screw-cap tube (previously washed and baked at 350 °C for 4 h) and spiked with 20 µL of IS solution. The sample was then mixed with 500 µL of acetate buffer (1 M, pH 5.0) containing approximately 200 active units of β-glucuronidase and heated overnight at 37 °C. After incubation, the sample was acidified with 300 µL of formic acid (HCOOH) and subjected to SPE.

The SPE sorbent was conditioned sequentially with 1 mL of ethyl acetate (EA), 1 mL of methanol (MeOH), and 1 mL of 1% HCOOH in water before loading the hydrolyzed urine sample. Then the sorbent was washed with 15% MeOH in 1% HCOOH in water (2 × 1 mL) and dried in the following two steps: (i) centrifugation for 2 min at 4000 rpm and (ii) drying under vacuum for 20 min. A two-step elution was conducted with EA (2 × 250 µL), the eluate was mixed with 10 µL of decane (keeper) and then evaporated to near dryness under a gentle stream of nitrogen at 35 °C. Decane (boiling point 174 °C), which does not interfere with silylating agents and GC analysis, was added to reduce the loss of dichlorophenols during the evaporation step. The dry residue was reconstituted with 50 µL of MSTFA and derivatized at 60 °C for 30 min. One microliter of the extract was injected into a GC/MS system.

### SPE method development

To achieve high extraction efficiency for all target analytes, the following parameters were studied: type of SPE sorbent, composition of washing solvent, and type and volume of the elution solvent. The method was developed using a pooled urine sample spiked with all analytes at a concentration of 20 ng mL^−1^. All experiments were repeated three times for each parameter, and results were compared based on the peak areas and relative standard deviation (RSD).

Three nonpolar polymeric SPE sorbents were tested: Agilent Bond Elut Plexa (30 mg, 1 mL), Supelco Supel-Select HLB (30 mg, 1 mL), and Waters OASIS HLB (60 mg, 3 mL). All sorbents are dedicated to extraction of a broad range of acidic, neutral, and basic compounds throughout the pH range of 1–14. Therefore, based on the general SPE protocols provided for each sorbent by the manufacturer, the extraction procedure was unified at this step and was as follows: the hydrolyzed sample was loaded onto a sorbent preconditioned with EA, MeOH, and 1% HCOOH in water, then the sorbent was washed with 10% MeOH in 1% HCOOH in water and dried under vacuum. The analytes were further eluted from the sorbent with MeOH.

After sorbent selection, the composition of the washing solvent was selected. The presence of potential interferences in the chromatograms and their influence on the recovery were evaluated after washing the sorbent with various concentrations of MeOH in 1% HCOOH in water (concentration range 10–40%). MeOH and EA were then tested as possible elution solvents. Eventually, the analytes were eluted sequentially with five portions of solvent (2 × 250 µL and 3 × 500 µL), and each fraction was collected into a separate glass tube, evaporated, and individually analyzed by GC/MS.

For clarity of visual perception, only results obtained for non-labeled analytes are shown in the figures, as similar results were found for their labeled analogs. All plots were generated using GraphPad Prism (GraphPad Software, San Diego, CA, USA).

### GC/MS/MS analysis

Analyses were carried out employing a Varian CP-3800 gas chromatograph coupled to a Varian-1177 split/splitless injector and a Varian-320 triple quadrupole mass spectrometer (Varian, Palo Alto, CA, USA). The extract (1 μL) was injected in splitless mode into the injector set at 290 °C. Separation was achieved on a VF-5 ms (Agilent, Middelburg, Netherlands) low-bleed capillary column (30 m × 0.25 mm ID, 0.25 μm film thickness, with an integrated 10 m guard column) using the following oven program temperature: 60 °C for 1 min; 60–130 °C (40 °C min^−1^); 130–230 °C (7 °C min^−1^); 230–300 °C (10 °C min^−1^); and 300 °C held for 10 min. Helium was used as the carrier gas at a constant flow rate of 1.0 mL min^−1^.

The mass spectrometer was operated in electron impact ionization mode (EI, 70 eV) with the filament current and electron multiplier voltage set at 50 μA and 1500 V, respectively. The temperatures of the manifold, the ion source, and the transfer line were 40, 230, and 290 °C, respectively. The pressure in the collision cell was set at 1.5 mTorr, and 99.9995% pure argon was used as the collision gas for MS/MS reactions. The mass spectrometer was operated in the multiple reaction monitoring (MRM) mode. MRM transitions and other data acquisition parameters are presented in Table S1.

### Method validation

Method validation was carried out based on the bioanalytical method validation guidelines provided by the Food and Drug Administration (FDA) [15]. Calibration and quality control samples were prepared by spiking 2 mL of urine with 40 µL of an appropriate working solution containing all target biomarkers and 20 µL of IS solution. To monitor contamination from reagents or materials used in the protocol, procedural blanks were always added to the analyzed urine samples. A procedural blank is a matrix-free sample treated in the same way as the test samples.

Nine-point matrix-matched calibration curves were prepared in the concentration range of 0.1–50 ng mL^−1^; however, for some biomarkers (MeP, EtP, PrP, BP-1, BP-3, and TCS), the upper concentration level was 1000 ng mL^−1^. All calibration curves were weighted (1/*x*) and presented as a plot of the ratio of analyte peak area to IS versus the ratio of analyte concentration to IS. The limit of quantification (LOQ) was defined as the lowest concentration of the calibration curve quantified with acceptable precision and accuracy.

Due to the wide range of calibration curves for the biomarkers mentioned above, quality control (QC) samples were prepared at five concentration levels: 1, 4, 12, 30, and 200 ng mL^−1^. A single validation run included six samples for each QC concentration (intra-day precision; *n* = 6), and analyses were repeated in three different validation runs (inter-day precision; *n* = 18). Precision was expressed as relative standard deviation (RSD), and accuracy was estimated as the ratio of the measured concentration to the nominal value. Results were accepted when the RSD was below 20% and accuracy was in the range of 80–120% of the nominal value. In addition, the quality of the method for BPA, BP-1, BP-3, TCS, 1-NP, 2-NP, TCPyr, and PCP was ensured by analyzing reference samples provided under the German External Quality Assessment Scheme (G-EQUAS).

## Results and discussion

### GC/MS/MS

**Fig. 1:**
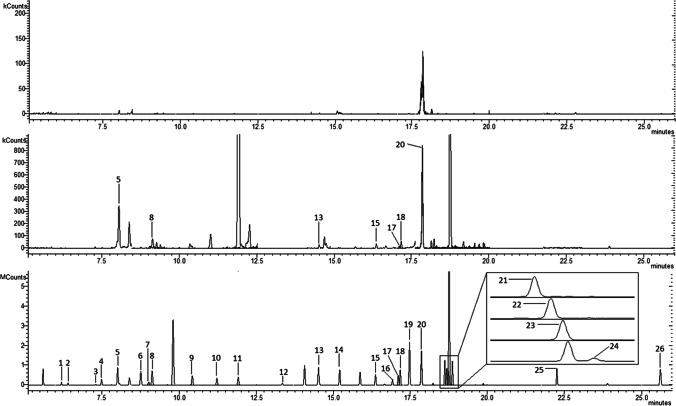
Extracted ion chromatogram of MSTFA (top), real urine sample (middle), and blank urine spiked at a known concentration of 4 ng mL^−1^ (bottom). 1: 2,5-DCP, 2: 2,4-DCP, 3: TCPyr, 4: 6-CNA, 5: MeP, 6: 1-NP, 7: EtP, 8: 2-NP, 9: PrP, 10: iBuP, 11: BuP, 12: PCP, 13: 3-PBA, 14: BPAF, 15: BP-3, 16: TCS, 17: BP-1, 18: BPF, 19: BPE, 20: BPA, 21: BPC, 22: 4OH3PBA, 23: BPB, 24: BPG, 25: BPS, 26: BPBP

After adjusting the column temperature program and defining retention times for each compound, the chromatogram was divided into 20 separate time segments. Each segment contained at most four compounds, and two MRM transitions were selected for each compound. The collision energy (CE) for each MRM transition was first tested in the range of 5–40 V (steps of 5 V) and then narrowed to the previously selected value ± 5 V (steps of 1 V). MRM transitions and other data acquisition parameters are shown in Table S1. Exemplary, extracted ion chromatograms of a blank, real sample, and spiked urine are shown in Fig. 1.

### SPE method development

#### SPE sorbent

Three hydrophilic styrene-based polymeric sorbents were investigated since they allowed the extraction of substances with a wide range of physicochemical properties. Based on the generic protocols provided by the manufacturers, one method was established for all three sorbents. As the OASIS HLB column had twice the amount of sorbent, the volume of all reagents was also doubled, except for 10% MeOH solvent used to wash the sorbent after sample loading. The sample volume was kept constant (3 mL).

**Fig. 2:**
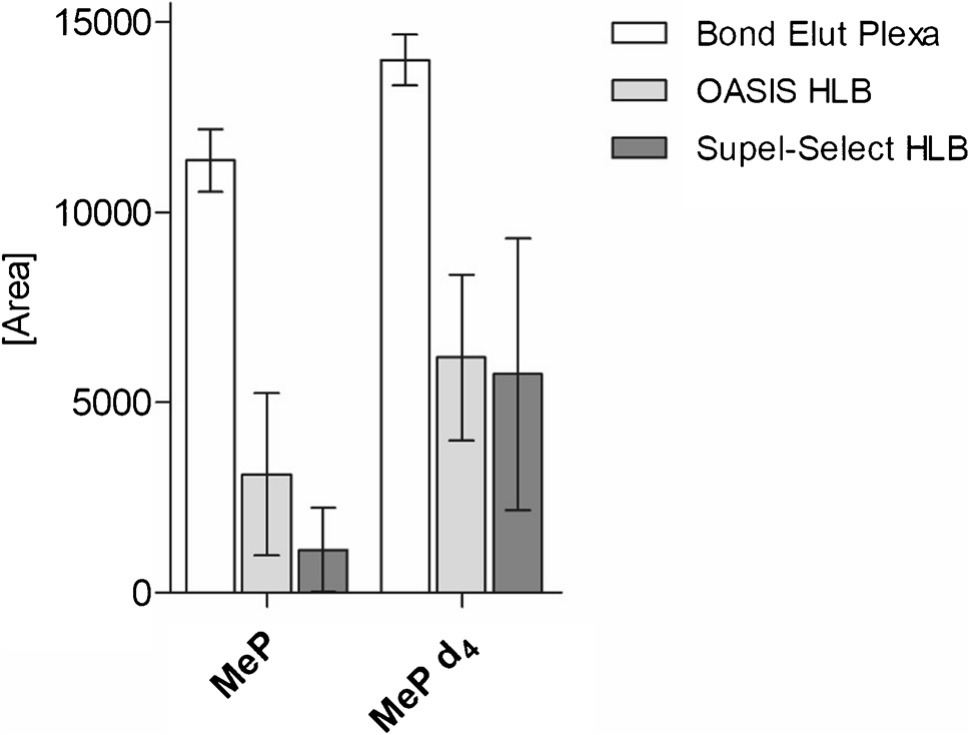
Comparison of the extraction efficiency of methyl paraben (MeP) and methyl paraben d_4_ (MeP-d_4_) in urine using three SPE sorbents

The highest repeatability was observed for Bond Elut Plexa (1.2–19.6%), while 9.9–68.6% was found for OASIS HLB and 1.5–99.0% for Supel-Select HLB. Higher and comparable peak areas were reported for Bond Elut Plexa and Supel-Select HLB, but lower for OASIS HLB (Fig. S1). However, the selection of Bond Elut Plexa was based on the results obtained for critical compounds: MeP and MeP-d_4_. We observed that the peak areas of MeP and MeP-d_4_ obtained for Bond Elut Plexa were at least twice as high as for OASIS HLB and Supel-Select HLB (Fig. 2). Although all three sorbents interact with chemicals based on a similar chemical mechanism, Bond Elut Plexa sorbent has an additional physical feature, which is a unique polymer structure consisting of a hydrophobic core and a hydrophilic amide-free surface. The polarity gradient on the polymer surface allows small molecules to penetrate into its interior, while the porous structure reduces the binding of matrix components, such as proteins, through a size exclusion mechanism [16]. Supel-Select HLB and OASIS HLB sorbents are universal reversed-phase sorbents made of hydrophilic, modified styrene-based polymers that provide enhanced retention of more polar chemicals [17, 18]. Binding of analytes to the core of the Plexa sorbent and its porous structure can result in stronger retention of target compounds while more efficiently removing interfering matrix components. As a result, these special properties may be beneficial for the repeatability of results and signals of some analytes, as observed for labeled and native MeP in this study.

#### Composition of the washing solvent

**Fig. 3:**
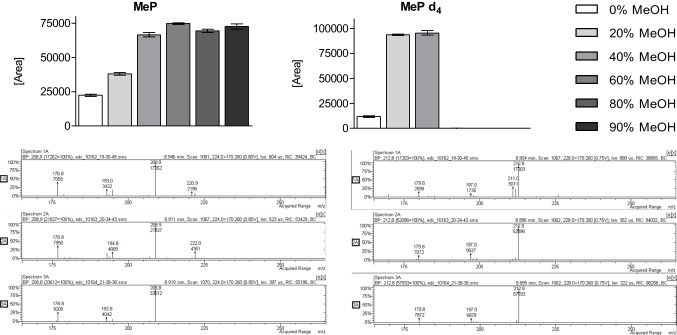
Investigation of the matrix effect in the frame of the washing solvent optimization step. The MS spectra represent samples after washing the sorbent with: 1% HCOOH in water (top, 1A); 20% MeOH in 1% HCOOH in water (middle, 2A); 40% MeOH in 1% HCOOH in water (bottom, 3A)

At the beginning of the SPE method development, 3 mL of urine was used, as previously described for the LLE method [19–21]. However, we noticed that the composition of the washing solvent significantly affected the absolute recovery of some analytes. The test washing compositions consisted of MeOH (concentration range 0–90%) and 1% HCOOH in water. It was found that the peak areas of MeP and MeP-d_4_ increased when the MeOH concentration increased from 0 to 40% (Fig. 3). Interestingly, washing the sorbent with the solvent containing > 40% MeOH did not affect the MeP response, but completely removed MeP-d_4_. Given these results, we hypothesized that the matrix could affect the response of these compounds, since a low concentration of MeOH could not remove excess matrix components that may further interfere with the GC system. On the other hand, a high concentration of MeOH could remove compounds of interest from the sorbent during the washing step.

The matrix effect was investigated using a separate GC/MS system equipped with an ion-trap mass spectrometer (Varian 220-MS, Varian, Palo Alto, CA, USA). In the ion trap, isolation and fragmentation of ions occurs in a small space (trap), which may be saturated by excess matrix components. Therefore, isolation/detection of required ions can be affected by the presence of matrix ions with similar *m/z* values in the trap. In such case, the sample preparation step is crucial to obtain reliable results. We observed that an increase in the percentage of MeOH in the washing solvent was associated with an increase in peak areas of MeP and MeP-d_4_, but also with a decline in the number of additional ions in MS spectra of retention times corresponding to both native and labeled MeP (Fig. 3).

Based on this experiment, we decided to reduce the sample volume from 3 to 2 mL and re-evaluate the composition of the washing solvent in the range of 10–40% MeOH in 1% HCOOH in water. For most analytes, there was no difference in response regardless of the composition tested. When the MeOH concentration was > 15%, the area of 6-CNA decreased, so the final composition was 15% MeOH in 1% HCOOH in water (Fig. S2). Methanol, due to its high eluting strength, removes from the sorbent either polar matrix components or more polar analytes such as 6-CNA. Therefore, the final composition of the washing solvent was a compromise between sufficient removal of impurities and satisfactory recovery of all analytes (60–113%).

#### Elution

According to general SPE protocols, MeOH is usually recommended for eluting analytes from a sorbent. MeOH is compatible with many SPE sorbents and analytes; however, it also elutes many polar matrix components. To reduce matrix interferences in the final extract, we compared MeOH and EA as elution solvents. Elution with EA generated a noticeably less colored extracts compared to MeOH, suggesting that more sample components were not eluted with EA. The peak areas of most analytes obtained for both solvents were similar; however, EA generated twice the response for BP-3 and BP3-d_5_ compared to MeOH (Fig. S3). Therefore, EA was incorporated into the final protocol.

The volume of EA was selected after performing sequential elution with five portions of the solvent (2 × 250 µL and 3 × 500 µL). Each fraction was collected into a separate glass tube, evaporated, and individually analyzed by GC/MS/MS. All analytes were recovered from the sorbent in the first two fractions, with more than 70% of all analytes recovered in the first fraction (Fig. S4).

### Validation

The results of method validation are presented in Table 1. Linearity was demonstrated throughout the working range of calibration curves for all analytes except 2,4-DCP, for which the upper calibration level was 20 ng mL^−1^. Correlation coefficients were > 0.99 (except for 6-CNA and BPBP), and LOQs were in the range of 0.1–0.25 ng mL^−1^. Higher LOQs were established for MeP and EtP (0.5 ng mL^−1^). Precision was in the range of 4.1–17.1%; however, lower reproducibility was reported for BPA (19.4%) and 6-CNA (19.8%) at the lowest QC level (Table S3). BPA was consistently present in the procedural blanks at an average concentration of 0.434 ng mL^−1^, and this background was subtracted from the BPA amount in the urine samples.

**Table 1:** Calibration parameters: internal standard, limit of quantification (LOQ), and calibration curve describing factors

	Analyte	IS	LOQ (ng mL^−1^)	Linear range (ng mL^−1^)	Regression equation	Regression coefficient (R)
1	MeP	MeP-d_4_	0.5	0.5–1000	y = 0.035x + 0.042	0.992
2	EtP	MeP-d_4_	0.5	0.5–1000	y = 0.007x + 0.003	0.999
3	PrP	PrP-^13^C_6_	0.25	0.25–1000	y = 0.034x + 0.003	0.998
4	iBuP	BuP-^13^C_6_	0.1	0.1–50	y = 0.016x—0.001	0.994
5	BuP	BuP-^13^C_6_	0.25	0.25–50	y = 0.020x + 0.002	0.996
6	BPA	BPA-d_16_	0.25	0.25–50	y = 0.055x + 0.066	0.998
7	BPAF	BPAF-d_4_	0.1	0.1–50	y = 0.054x—0.001	0.996
8	BPB	BPA-d_16_	0.1	0.1–50	y = 0.051x + 0.003	0.998
9	BPBP	BPA-d_16_	0.1	0.1–50	y = 0.031x—0.002	0.987
10	BPC	BPA-d_16_	0.1	0.1–50	y = 0.040x—0.001	0.998
11	BPE	BPA-d_16_	0.1	0.1–50	y = 0.062x—0.001	0.998
12	BPF	BPF-d_10_	0.25	0.25–50	y = 0.097x + 0.001	0.997
13	BPG	BPA-d_16_	0.1	0.1–50	y = 0.025x—0.001	0.997
14	BPS	BPS-d_8_	0.1	0.1–50	y = 0.031x—0.002	0.998
15	BP-1	BP3-d_5_	0.25	0.25–1000	y = 0.033x + 0.019	0.999
16	BP-3	BP3-d_5_	0.25	0.25–1000	y = 0.029x + 0.004	0.998
17	TCS	TCS-d_3_	0.25	0.25–1000	y = 0.011x + 0.005	0.997
18	2,4-DCP	2,4-DCP-d_3_	0.1	0.1–20	y = 0.060x + 0.003	0.997
19	2,5-DCP	2,4-DCP-d_3_	0.1	0.1–50	y = 0.053x—0.001	0.996
20	PCP	BP3-d_5_	0.5	0.5–50	y = 0.007x—0.001	0.974
21	TCPyr	TCPyr-^13^C_3_	0.25	0.25–50	y = 0.090x + 0.009	0.998
22	6-CNA	MeP-d_4_	0.25	0.25–50	y = 0.019x—0.001	0.986
23	1-NP	1-NP-d_7_	0.25	0.25–50	y = 0.177x + 0.005	0.993
24	2-NP	2-NP-d_7_	0.25	0.25–50	y = 0.107x + 0.070	0.998
25	3-PBA	2-PBA	0.25	0.25–50	y = 0.048x + 0.014	0.998
26	4OH3PBA	2-PBA	0.25	0.25–50	y = 0.040x + 0.004	0.998

Accuracy was also satisfactory, ranging from 78 to 118% for most analytes. Lower accuracy (< 70%) was observed at the lowest QC level for 6-CNA, since this analyte can be partially removed from the sorbent during the washing step, as shown in Fig. S2. MeOH concentration above 15% reduced the response of 6-CNA. In contrast, consistently higher concentrations than nominal values (accuracy: 106–134%) were observed for more lipophilic bisphenols (logP > 5.0), such as BPC, BPG, and BPBP. However, due to their physicochemical properties, the question should be asked whether a parent compound (BPBP, BPC, and BPG) is a suitable biomarker for determination in urine. Controlled toxicokinetics studies are needed to answer this question. The quality control test also was not successful for PCP, for which low accuracy (56–84%) and precision > 15% were reported. The results obtained for analytes present in the G-EQUAS samples were within the reference range at both concentration levels (Table S2). Finally, a total of 22 biomarkers met all validation criteria.

### Storage stability of biomarkers in urine

Non-persistent chemicals are usually excreted in urine as a mixture of conjugates with various endogenous substrates. In HBM studies, the total concentration of the compound (unconjugated plus conjugated species) after hydrolysis of the conjugate(s) is usually determined to estimate internal exposure. It is worth noting that the presence of the free form of some compounds (e.g., parabens, BPA) may be due to external contamination of the sample, as it is well documented that they are excreted almost exclusively in conjugated form. Only a few studies have been published investigating the stability of phenolic compounds in urine samples [12, 13, 22], two of which examined the stability of conjugated forms [12, 13].

Urine biomarker concentrations measured on the day of collection (D0) are shown in Table 2. Since our objective was also to investigate the stability during the first 24 h after collection, it was crucial to collect a fresh urine sample containing incurred concentrations of investigated biomarkers. For this purpose, we recruited one female volunteer who used numerous personal care products, including make-up products and cosmetics. Random morning spot samples were previously screened for the presence of compounds of interest. Prior to the day of collection, a female volunteer was asked to maintain a daily routine to ensure a high level of selected biomarkers (MeP, EtP, PrP, BuP, BP-3). The collected sample was additionally spiked with glucuronides of three bisphenols (BPA, BPF, and BPS), as the background level was considered too low for stability testing.

**Table 2:** Total concentrations (sum of conjugated and free form) and percentage of free form in urine sample for selected biomarkers at final time points. Samples were stored at room temperature (RT), 4 °C, and −20 °C

Analyte	D0		D3 (RT)		M2 (4 °C)		M18 (−20 °C)		*M36 (−20 °C)*^*a*^	
	ng mL^−1^	Free (%)	ng mL^−1^	Free (%)	ng mL^−1^	Free (%)	ng mL^−1^	Free (%)	*ng mL*^*−1*^	*Free (%)*
MeP	127.6	0.7	136.7	37.9	155.9	5.7	131.3	1.7	*94.1*	*3.4*
EtP	136.3	0.4	138.0	47.1	139.3	3.8	119.3	0.9	*145.8*	*4.5*
PrP	139.5	0.2	140.6	31.7	133.1	3.3	132.7	1.1	*115.7*	*8.1*
BuP	22.9	1.0	21.8	22.4	22.2	3.6	20.6	2.4	*18.5*	*3.1*
BPA	12.4	0.0	11.4	3.8	12.4	2.8	12.6	0.0	*9.20*	*0.0*
BPF	14.9	0.0	11.8	26.4	15.9	1.8	13.0	0.0	*10.6*	*0.0*
BPS	5.78	0.0	5.40	4.3	6.04	5.8	5.43	0.0	*9.27*	*0.0*
BP-3	16.2	4.0	15.9	3.9	16.0	1.8	14.6	1.7	*11.1*	*10.0*
2,4-DCP	0.478	0.0	0.827	21.0	0.764	0.0	0.505	0.0	*0.550*	*0.0*
2-NP	1.49	0.0	1.41	60.7	1.26	0.0	1.29	0.0	*1.13*	*0.0*
BP-1	nd	nd	nd	nd	nd	nd	nd	nd	*5.28*	*0.0*

^a^(*italics*) – samples were analyzed using a changed protocol than previous samples (D0, D3, M2, and M18)

nd – not reported in this study

Ye et al. examined the stability of eight phenols (MeP, EtP, PrP, BuP, BPA, BP-3, TCS, and 2,5-DCP) in urine samples stored at three different temperatures (RT, 4 °C and −70 °C) [13]. The authors found that some analytes started to degrade within 24 h after collection when stored at RT (BPA, BP-3), while all conjugates were stable at −70 °C for 180 days and at 4 °C for at least 7 days. However, not all laboratories are capable of storing samples at such low temperature (−70 °C); thus, storing samples at −20 °C in many cases is more feasible. Also, in some HBM studies, the participants collected and froze samples at home [23]. Therefore, we investigated the stability of biomarkers in urine samples stored at −20 °C for up to 18 months. The storage time at a specific temperature was selected on the basis of the probable storage conditions of real samples in HBM studies.

All conjugates stored at −20 °C were stable throughout the study period. Low concentrations of free MeP, EtP, PrP, BuP, and BP-3 were quantified in samples analyzed without hydrolysis; however, they accounted for < 2.5% of the concentrations in enzymatically treated samples. The total concentration of all biomarkers did not change significantly when the samples were stored at −20 °C for 18 months. Parabens began to degrade within 48 h (D2) in samples stored at 4 °C; however, at the end of the experiment concentration of free form accounted for < 6% of the total concentration. Interestingly, the concentration of free BP-3 decreased from 0.649 ng mL^−1^ to 0.285 ng ml^−1^, while for free 2,4-DCP doubled over the same period (from 0.478 ng mL^−1^ to 0.764 ng mL^−1^). It was also observed that the total MeP concentration increased from 127.6 ng mL^−1^ (D0) to 155.9 ng mL ^−1^ on day 7 (D7) in samples stored in a refrigerator. Given the aforementioned matrix effect observed for MeP, this increase in the concentration may be due to changes that occurred in the matrix rather than in the concentration of the biomarker. However, more experiments with a greater number and diversity of urine samples should be performed to explain this phenomenon. Nevertheless, the greatest changes were observed for samples stored at RT. All paraben conjugates began to degrade (hydrolyze) within 48 h after collection, and on day 3 (D3) the concentration of their free form accounted for 22–47% of the total concentration. A similar increase was observed for 2,4-DCP (21%), BPF (26%), and 2-NP (61%), while for the remaining compounds the concentrations were stable or constantly < LOQ.

Despite the increase in the free form concentration, the total concentrations of most biomarkers did not change significantly. The exception was 2,4-DCP, as its total concentration doubled on D3. The results of the stability test are presented in Table 2, but also in Fig. S5 and Fig. S6. The main study protocol included a stability study for 18 months; however, some urinary aliquots still remained after this time. Therefore, an additional analysis was performed after 36 months (36 M). Since the entire volume of frozen aliquots was less than 10 mL, the analyses were performed in triplicate using 1 mL of urine. Although the urine volume was reduced compared to the validated method, we did not change any of the other steps in the sample preparation protocol. As described in Sect. 3.2, the amount of matrix retained on the sorbent and the composition of the washing solvent can significantly affect the recovery of some analytes; these results were used to investigate the stability of conjugates and should be carefully interpreted when comparing the stability of the total concentrations. All conjugates were stable after 36 M when stored at −20 °C. The concentration of free forms in samples analyzed without hydrolysis was < 5% of the total concentration, except for PrP (8.1%) and BP-3 (10.0%). The results are shown in Table 2.

#### Freeze–thaw cycles

**Fig. 4:**
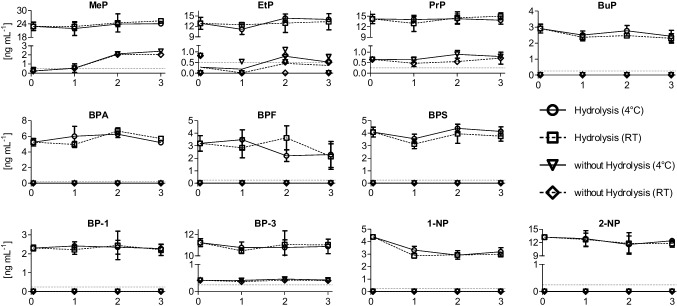
Urine sample stability over three freeze–thaw cycles. Followed by thawing at room temperature for a few hours (room temp) or in a refrigerator overnight (4 °C), samples were analyzed without (without Hydrolysis) or after hydrolysis (Hydrolysis). Number 0 represents the concentration of biomarkers in the fresh urine sample, and numbers 1–3 represent consecutive freeze–thaw cycles

The stability of the conjugates was investigated after three freeze–thaw cycles, with a minimum of 1 week between cycles. During each cycle, samples were thawed in a dark cabinet at RT or in a refrigerator at 4 °C. The results are shown in Fig. 4. We found that total and conjugate concentrations were stable for most of the biomarkers, except MeP and 1-NP. The concentration of free MeP gradually increased from < LOQ in the fresh sample to > 2 ng mL^−1^ in cycle 2. However, the total concentration did not change, regardless of the thawing conditions.

The total concentration of 1-NP dropped by 25% in the first and 30% after the third thawing cycle, but the free form concentration was always < LOQ. The decrease in total concentration was observed regardless of the thawing conditions and was statistically significant (one-way analysis of variance, *p* < 0.001). Unlike 1-NP, the total concentration of 2-NP did not differ after the third cycle (one-way analysis of variance, *p* > 0.5). Limited stability of 1-NP in urine samples was also observed in other studies [12, 22]. Gaudreau et al. observed a decrease in 1-NP conjugate and free form concentrations when samples were stored at RT. The concentration was < LOD after 16 weeks, and the entire study period was 1 year [12]. Hoppin et al. investigated the stability of several chemicals by spiking urine with a standard solution and noticed a degradation of 1-NP, even when preservatives were added to the sample. Moreover, changes were observed regardless of the type of preservative [22].

All biomarkers were stable under typical storage conditions: RT for 24 h, 4 °C for 7 days, and −20 °C for 18 months. Importantly, the measurement of the total concentration of the given biomarkers was not affected by thawing conditions. A limitation of this study, however, is that the stability assessment was conducted using a single urine sample, and thus it was not possible to examine the impact of matrix effects on the results.

### Application of the method

The validated method was used to analyze 38 randomly selected 24-h urine samples collected within a study described elsewhere [23]. Ten out of 26 biomarkers were detected in more than 50% of samples (Table S4). 2-NP was detected in all samples, while MeP, EtP, PrP, BuP, BPA, and TCPyr were detected in more than 80% of the samples. The highest median concentration was estimated for MeP (18.9 ng mL^−1^). BPF and BPS were quantified in only 20% of the samples, whereas other bisphenols were not detected in any sample. These results are in line with those obtained in other European countries (Table S4). These analyses confirmed the applicability of the method in biomonitoring studies, making it suitable for future use in assessing the exposure of the general population to selected non-persistent environmental pollutants and supporting the human health risk assessment.

## Conclusions

A method for analyzing biomarkers of selected environmental chemicals to assess the exposure to 26 parent compounds in human urine has been developed. Overall, 22 biomarkers were quantified with satisfactory accuracy and precision. Validation criteria were not met for more lipophilic compounds (logP > 5.0), such as BPBP, BPC, and BPG. Due to the use of commercially available sorbents, the method can be easily applied in most laboratories and does not require additional equipment as in the case of online SPE. Moreover, it is characterized by comparable analytical performance to previously published methods (Table S5), supporting its applicability in future HBM studies. Our work also provides new information on the stability of biomarkers in urine samples stored and thawed under different conditions. We found that the total concentrations of all biomarkers studied were stable for at least 18 months when urine samples were stored at −20 °C. Moreover, the results of this study showed that thawing conditions do not affect the stability of conjugates. The limited stability of 1-NP in urine was consistent with previous studies.

## Supplementary Information

Below is the link to the electronic supplementary material.

Supplementary file 1 (DOCX 2308 KB)

## Data Availability

All data generated or analyzed during this study are included in this published article and its supplementary information file.
