# Supplementary material for: Quantification and stability assessment of urinary phenolic and acidic biomarkers of non-persistent chemicals using the SPE-GC/MS/MS method
Source: Anal Bioanal Chem. 2023 Mar 18;415(12):2227–38. doi: 10.1007/s00216-023-04633-7 (PMC10115689; doi:10.1007/s00216-023-04633-7)
Supplement: Supplementary file 1 — (DOCX 2308 KB) [file 216_2023_4633_MOESM1_ESM.docx]

Supplementary Information

**Quantification and stability assessment of urinary phenolic and acidic biomarkers of non-persistent chemicals using SPE-GC/MS/MS method**

Authors: Anna Klimowska ^1^, Evelien Wynendaele ^2^, Bartosz Wielgomas ^1,*^

^1^ Department of Toxicology, Faculty of Pharmacy, Medical University of Gdańsk, 107 Hallera Street, 80-416 Gdańsk, Poland

^2^ DruQuaR laboratory, Faculty of Pharmaceutical Sciences, Ottergemse steenweg 460, B 9000 Gent, Belgium

* -Bartosz Wielgomas – **corresponding author**

E-mail: bartosz.wielgomas@gumed.edu.pl

**Table S1** GC-QqQ-MS/MS data acquisition parameters for all target analytes and internal standards.

| Analyte | Acronym | Time segment | Retention time (min) | MRM transitions, *m/z* (collision energy, eV) | |
| --- | --- | --- | --- | --- | --- |
|  |  |  |  | Quantitative | Qualitative |
| 2,5-Dichlorophenol | 2,5-DCP | 1 | 7.432 | 234 > 219 (5) | 219 > 93 (9) |
| 2,4-Dichlorophenol d_3_ | 2,4-DCP-d_3_ | 1 | 7.624 | 222 > 93 (9) | 237 > 222 (10) |
| 2,4-Dichlorophenol | 2,4-DCP | 1 | 7.656 | 234 > 219 (5) | 219 > 93 (9) |
| Trichloro-2-pyridinol ^13^C_3_ | TCPyr-^13^C_3_ | 2 | 8.524 | 257 > 93 (6) | 257 > 222 (7) |
| Trichloro-2-pyridinol | TCPyr | 2 | 8.529 | 254 > 93 (10) | 254 > 219 (5) |
| 6-Chloronicotinic acid | 6-CNA | 3 | 8.695 | 214 > 167 (5) | 214 > 140 (14) |
| Methyl paraben d4 | MeP-d_4_ | 4 | 9.144 | 228 > 213 (5) | 213 > 180 (5) |
| Methyl paraben | MeP | 4 | 9.169 | 224 > 209 (5) | 209 > 177 (5) |
| 1-Naphthol d_7_ | 1-NP-d_7_ | 5 | 9.898 | 223 > 208 (7) | 208 > 191 (7) |
| 1-Naphthol | 1-NP | 5 | 9.946 | 216 > 201 (5) | 201 > 185 (7) |
| Ethyl paraben | EtP | 6 | 10.132 | 238 > 223 (6) | 223 > 151 (6) |
| 2-Naphthol d_7_ | 2-NP-d_7_ | 6 | 10.253 | 223 > 208 (7) | 208 > 191 (7) |
| 2-Naphthol | 2-NP | 6 | 10.296 | 216 > 201 (7) | 201 > 185 (7) |
| Propyl paraben | PrP | 7 | 11.538 | 210 > 195 (6) | 252 > 210 (5) |
| Propyl paraben ^13^C_6_ | PrP-^13^C_6_ | 7 | 11.543 | 216 > 201 (5) | 258 > 216 (4) |
| iso-Butyl paraben | iBuP | 8 | 12.337 | 210 > 195 (5) | 266 > 210 (5) |
| Butyl paraben ^13^C_6_ | BuP-^13^C_6_ | 9 | 13.032 | 216 > 201 (5) | 272 > 216 (5) |
| Butyl paraben | BuP | 9 | 13.035 | 210 > 195 (6) | 266 > 210 (5) |
| 2-Phenoxybenzoic acid | 2-PBA | 10 | 14.370 | 286 >193 (5) | 271 > 211 (6) |
| Pentachlorophenol | PCP | 10 | 14.519 | 323 > 93 (14) | 338 > 323 (6) |
| 3-Phenoxybenzoic acid | 3-PBA | 11 | 15.630 | 271 > 227 (6) | 286 > 271 (6) |
| Bisphenol AF d_4_ | BPAF-d_4_ | 12 | 16.300 | 484 > 415 (9) | 469 > 317 (7) |
| Bisphenol AF | BPAF | 12 | 16.330 | 480 > 411 (8) | 465 > 315 (7) |
| Benzophenone-3 d_5_ | BP3-d_5_ | 13 | 17.468 | 290 > 247 (9) | 305 > 290 (5) |
| Benzophenone-3 | BP-3 | 13 | 17.490 | 285 > 242 (12) | 300 > 285 (5) |
| Triclosan d_3_ | TCS-d_3_ | 14 | 18.008 | 350 > 200 (10) | 363 > 348 (6) |
| Triclosan | TCS | 14 | 18.019 | 347 > 200 (10) | 360 > 345 (6) |
| Bisphenol F d_10_ | BPF-d_10_ | 15 | 18.182 | 354 > 185 (17) | 354 > 73 (11) |
| Benzophenone-1 | BP-1 | 15 | 18.183 | 343 > 271 (9) | 358 > 343 (5) |
| Bisphenol F | BPF | 15 | 18.240 | 329 > 179 (12) | 344 > 179 (7) |
| Bisphenol E | BPE | 16 | 18.540 | 358 > 343 (6) | 343 > 73 (15) |
| Bisphenol A d_16_ | BPA-d_16_ | 17 | 18.859 | 368 > 197 (13) | 386 > 368 (6) |
| Bisphenol A | BPA | 17 | 18.942 | 357 > 191 (13) | 372 > 357 (6) |
| Bisphenol C | BPC | 18 | 19.650 | 385 > 205 (11) | 400 > 385 (8) |
| 4-Hydroxy-3-phenoxybenzoic acid | 4OH3PBA | 18 | 19.712 | 374 > 359 (6) | 359 > 315 (5) |
| Bisphenol B | BPB | 18 | 19.780 | 357 > 191 (8) | 386 > 357 (7) |
| Bisphenol G | BPG | 18 | 19.899 | 441 > 233 (14) | 456 > 441 (6) |
| Bisphenol S d_8_ | BPS-d_8_ | 19 | 23.210 | 402 > 387 (9) | 387 > 233 (6) |
| Bisphenol S | BPS | 19 | 23.240 | 394 > 379 (7) | 379 > 229 (6) |
| Bisphenol BP | BPBP | 20 | 27.148 | 496 > 419 (6) | 419 > 253 (7) |

**Table S2** External quality assessment – results of G-EQUAS samples from round 67/2021

|  | 14/15 A (ng mL^-1^) | | 14/15 B (ng mL^-1^) | |
| --- | --- | --- | --- | --- |
|  | Result | Reference value (range) | Result | Reference value (range) |
| TCPyr | 1.01 | 1.28 (0.95 – 1.62) | 6.91 | 8.51 (6.20 – 10.82) |
| 1-NP | 2.51 | 2.58 (1.77 – 3.39) | 12.28 | 10.97 (7.97 – 13.97) |
| 2-NP | 3.65 | 3.74 (2.72 – 4.76) | 18.41 | 19.64 (15.47 – 23.81) |
| PCP | 2.17 | 1.74 (1.26 – 2.22) | 6.76 | 6.08 (4.58 – 7.58) |
| BP-1 | 2.60 | 2.75 (2.00 – 3.50) | 5.62 | 5.19 (4.14 – 6.24) |
| BP-3 | 3.39 | 4.27 (3.13 – 5.41) | 8.11 | 9.60 (7.59 – 11.61) |
| TCS | 9.22 | 11.4 (8.1 – 14.7) | 45.9 | 54.5 (39.5 – 69.5) |
| BPA | 0.48 | 0.58 (0.37 – 0.79) | 11.69 | 13.83 (10.47 – 17.19) |

**Table S3** Quality control – intraday (n=6) and intraday (n=18) precision and accuracy

|  | Analyte | 1 ng mL^-1^ | | | | 4 ng mL^-1^ | | | | 12 ng mL^-1^ | | | | 30 ng mL^-1^ | | | | 200 ng mL^-1^ | | | |
| --- | --- | --- | --- | --- | --- | --- | --- | --- | --- | --- | --- | --- | --- | --- | --- | --- | --- | --- | --- | --- | --- |
|  |  | D1  (n=6) | D2  (n=6) | D3  (n=6) | **D1-3**  **(n=18)** | D1 | D2 | D3 | **D1-3** | D1 | D2 | D3 | **D1-3** | D1 | D2 | D3 | **D1-3** | D1 | D2 | D3 | **D1-3** |
| 1 | **MeP** |  |  |  |  |  |  |  |  |  |  |  |  |  |  |  |  |  |  |  |  |
|  | AM (ng mL^-1^) | 0.927 | 0.785 | 0.826 | **0.846** | 4.00 | 3.41 | 3.50 | **3.64** | 12.5 | 11.0 | 10.7 | **11.4** | 31.5 | 26.8 | 27.4 | **28.6** | 203 | 191 | 187 | **193** |
|  | Accuracy (%) | 92.7 | 78.5 | 82.6 | **84.6** | 100 | 85.1 | 87.4 | **90.9** | 104 | 91.7 | 89.4 | **95.1** | 105 | 89.4 | 91.2 | **95.3** | 102 | 95.7 | 93.6 | **96.4** |
|  | RSD (%) | 17.4 | 11.8 | 14.5 | **15.8** | 14.1 | 10.1 | 8.2 | **13.0** | 12.0 | 7.9 | 8.4 | **11.6** | 10.9 | 6.4 | 10.0 | **11.7** | 5.7 | 7.0 | 4.6 | **6.5** |
| 2 | **EtP** |  |  |  |  |  |  |  |  |  |  |  |  |  |  |  |  |  |  |  |  |
|  | AM (ng mL^-1^) | 0.946 | 0.953 | 1.06 | **0.985** | 3.99 | 4.68 | 4.73 | **4.47** | 12.3 | 13.9 | 12.6 | **12.9** | 32.7 | 34.2 | 35.2 | **33.8** | 198 | 221 | 215 | **210** |
|  | Accuracy (%) | 94.6 | 95.3 | 106 | **98.5** | 99.8 | 117 | 118 | **112** | 102 | 116 | 105 | **108** | 109 | 114 | 117 | **113** | 99.0 | 110 | 107 | **105** |
|  | RSD (%) | 10.0 | 16.2 | 21.2 | **16.3** | 13.0 | 11.1 | 8.7 | **12.8** | 7.3 | 11.6 | 6.3 | **10.2** | 3.1 | 9.2 | 5.8 | **6.6** | 7.7 | 9.7 | 12.8 | **10.6** |
| 3 | **PrP** |  |  |  |  |  |  |  |  |  |  |  |  |  |  |  |  |  |  |  |  |
|  | AM (ng mL^-1^) | 0.929 | 1.06 | 1.01 | **0.998** | 3.78 | 4.35 | 4.08 | **4.07** | 11.4 | 13.1 | 12.9 | **12.5** | 28.5 | 33.3 | 35.2 | **32.1** | 179 | 216 | 217 | **205** |
|  | Accuracy (%) | 92.9 | 106 | 101 | **99.8** | 94.4 | 109 | 102 | **102** | 95.2 | 109 | 108 | **104** | 95.2 | 111 | 117 | **107** | 89.6 | 108 | 109 | **102** |
|  | RSD (%) | 11.3 | 12.2 | 8.7 | **11.4** | 9.8 | 7.4 | 10.7 | **10.5** | 4.0 | 7.9 | 8.7 | **9.2** | 8.9 | 8.8 | 8.2 | **12.2** | 5.4 | 8.6 | 9.5 | **11.8** |
| 4 | **iBuP** |  |  |  |  |  |  |  |  |  |  |  |  |  |  |  |  |  |  |  |  |
|  | AM (ng mL^-1^) | 0.781 | 0.881 | 0.966 | **0.876** | 3.47 | 3.87 | 4.18 | **3.84** | 11.0 | 12.3 | 12.5 | **11.9** | 28.3 | 32.5 | 34.5 | **31.8** |  |  |  |  |
|  | Accuracy (%) | 78.1 | 88.1 | 96.6 | **87.6** | 86.6 | 96.7 | 105 | **96.0** | 91.3 | 103 | 105 | **99,5** | 94.4 | 108 | 115 | **106** |  |  |  |  |
|  | RSD (%) | 10.4 | 13.8 | 10.8 | **14.2** | 11.8 | 6.1 | 12.6 | **12.7** | 9.2 | 4.9 | 8.9 | **9.5** | 7.4 | 10.1 | 7.4 | **11.5** |  |  |  |  |
| 5 | **BuP** |  |  |  |  |  |  |  |  |  |  |  |  |  |  |  |  |  |  |  |  |
|  | AM (ng mL^-1^) | 0.881 | 1.02 | 1.05 | **0.978** | 3.57 | 3.92 | 4.06 | **3.82** | 10.9 | 12.3 | 12.0 | **11.8** | 27.6 | 33.3 | 34.9 | **31.9** |  |  |  |  |
|  | Accuracy (%) | 88.1 | 102 | 105 | **97.8** | 89.1 | 98.1 | 102 | **95.6** | 91.1 | 103 | 100 | **97.9** | 91.9 | 111 | 116 | **106** |  |  |  |  |
|  | RSD (%) | 9.7 | 11.7 | 12.1 | **13.1** | 7.7 | 6.6 | 8.7 | **9.0** | 5.4 | 5.7 | 7.5 | **7.9** | 6.7 | 10.4 | 7.8 | **13.0** |  |  |  |  |
| 6 | **BPA** |  |  |  |  |  |  |  |  |  |  |  |  |  |  |  |  |  |  |  |  |
|  | AM (ng mL^-1^) | 0.840 | 0.856 | 0.834 | **0.844** | 3.60 | 3.85 | 3.82 | **3.78** | 11.5 | 12.3 | 11.6 | **11.8** | 29.6 | 30.2 | 31.0 | **30.3** |  |  |  |  |
|  | Accuracy (%) | 84.0 | 85.6 | 83.4 | **84.4** | 90.0 | 96.3 | 95.4 | **94.4** | 96.2 | 102 | 96.5 | **98.6** | 98.8 | 101 | 103 | **101** |  |  |  |  |
|  | RSD (%) | 19.8 | 20.1 | 22.0 | **19.4** | 5.7 | 10.3 | 7.8 | **8.5** | 3.6 | 6.0 | 7.5 | **6.5** | 5.1 | 6.4 | 4.0 | **5.2** |  |  |  |  |
| 7 | **BPAF** |  |  |  |  |  |  |  |  |  |  |  |  |  |  |  |  |  |  |  |  |
|  | AM (ng mL^-1^) | 0.937 | 0.921 | 0.908 | **0.922** | 4.03 | 4.05 | 3.96 | **4.01** | 12.1 | 12.6 | 12.1 | **12.2** | 30.9 | 32.0 | 32.3 | **31.8** |  |  |  |  |
|  | Accuracy (%) | 93.7 | 92.1 | 90.8 | **92.2** | 101 | 101 | 98.9 | **100** | 101 | 105 | 101 | **102** | 103 | 107 | 108 | **106** |  |  |  |  |
|  | RSD (%) | 5.0 | 8.9 | 6.5 | **6.7** | 3.7 | 8.4 | 4.6 | **5.7** | 2.4 | 5.0 | 4.9 | **4.6** | 2.8 | 7.9 | 4.7 | **5.6** |  |  |  |  |
| 8 | **BPB** |  |  |  |  |  |  |  |  |  |  |  |  |  |  |  |  |  |  |  |  |
|  | AM (ng mL^-1^) | 0.949 | 1.04 | 0.958 | **0.979** | 4.12 | 4.14 | 3.91 | **4.06** | 12.5 | 12.2 | 11.7 | **12.1** | 32.4 | 31.8 | 30.7 | **31.6** |  |  |  |  |
|  | Accuracy (%) | 94.9 | 104 | 95.8 | **97.9** | 103 | 104 | 97.6 | **101** | 104 | 102 | 97.4 | **101** | 108 | 106 | 103 | **106** |  |  |  |  |
|  | RSD (%) | 10.6 | 14.5 | 11.9 | **12.3** | 8.1 | 7.5 | 5.9 | **7.4** | 10.2 | 9.3 | 5.4 | **8.5** | 6.3 | 8.8 | 7.8 | **7.6** |  |  |  |  |
| 9 | **BPBP** |  |  |  |  |  |  |  |  |  |  |  |  |  |  |  |  |  |  |  |  |
|  | AM (ng mL^-1^) | 0.941 | 1.17 | 1.08 | **1.06** | 4.19 | 5.34 | 4.71 | **4.71** | 12.9 | 17.6 | 15.2 | **15.2** | 33.2 | 45.1 | 42.6 | **40.2** |  |  |  |  |
|  | Accuracy (%) | 94.1 | 117 | 108 | **106** | 105 | 134 | 118 | **118** | 108 | 147 | 127 | **127** | 111 | 151 | 142 | **134** |  |  |  |  |
|  | RSD (%) | 11.7 | 13.3 | 14.1 | **15.4** | 11.9 | 4.7 | 9.5 | **13.5** | 11.3 | 11.2 | 13.0 | **17.1** | 13.9 | 13.4 | 12.7 | **18.2** |  |  |  |  |
| 10 | **BPC** |  |  |  |  |  |  |  |  |  |  |  |  |  |  |  |  |  |  |  |  |
|  | AM (ng mL^-1^) | 1.17 | 1.18 | 1.30 | **1.21** | 5.02 | 5.07 | 5.44 | **5.18** | 14.5 | 15.3 | 15.0 | **14.9** | 33.8 | 34.1 | 35.8 | **34.6** |  |  |  |  |
|  | Accuracy (%) | 117 | 118 | 130 | **121** | 125 | 127 | 136 | **129** | 121 | 127 | 125 | **124** | 113 | 114 | 119 | **115** |  |  |  |  |
|  | RSD (%) | 18.0 | 4.9 | 5.2 | **11.6** | 7.8 | 6.5 | 4.2 | **6.9** | 8.9 | 5.8 | 1.2 | **6.3** | 10.5 | 6.6 | 4.9 | **7.6** |  |  |  |  |
| 11 | **BPE** |  |  |  |  |  |  |  |  |  |  |  |  |  |  |  |  |  |  |  |  |
|  | AM (ng mL^-1^) | 0.867 | 0.891 | 0.886 | **0.882** | 3.94 | 3.93 | 3.89 | **3.92** | 11.8 | 12.3 | 11.6 | **11.9** | 29.9 | 30.9 | 31.4 | **30.7** |  |  |  |  |
|  | Accuracy (%) | 86.7 | 89.1 | 88.6 | **88.2** | 98.5 | 98.1 | 97.4 | **98.0** | 98.5 | 102 | 97.0 | **99.3** | 99.6 | 103 | 105 | **102** |  |  |  |  |
|  | RSD (%) | 5.8 | 6.9 | 5.6 | **5.9** | 3.4 | 5.4 | 5.8 | **4.7** | 4.2 | 6.7 | 7.1 | **6.2** | 6.8 | 7.5 | 4.1 | **6.2** |  |  |  |  |
| 12 | **BPF** |  |  |  |  |  |  |  |  |  |  |  |  |  |  |  |  |  |  |  |  |
|  | AM (ng mL^-1^) | 0.955 | 1.00 | 0.952 | **0.969** | 3.97 | 3.75 | 3.73 | **3.82** | 12.1 | 11.6 | 10.9 | **11.6** | 30.5 | 29.9 | 29.6 | **30.0** |  |  |  |  |
|  | Accuracy (%) | 95.5 | 100 | 95.2 | **96.9** | 99.2 | 93.8 | 93.3 | **95.4** | 101 | 96.8 | 91.4 | **96.4** | 102 | 99.8 | 98.8 | **100** |  |  |  |  |
|  | RSD (%) | 5.4 | 11.4 | 9.3 | **8.6** | 5.6 | 12.9 | 9.0 | **9.4** | 7.0 | 6.1 | 6.5 | **7.5** | 5.4 | 7.7 | 8.8 | **7.0** |  |  |  |  |
| 13 | **BPG** |  |  |  |  |  |  |  |  |  |  |  |  |  |  |  |  |  |  |  |  |
|  | AM (ng mL^-1^) | 1.10 | 1.23 | 1.19 | **1.17** | 5.11 | 5.07 | 5.30 | **5.16** | 14.7 | 15.8 | 15.6 | **15.4** | 33.3 | 36.5 | 35.8 | **35.2** |  |  |  |  |
|  | Accuracy (%) | 110 | 123 | 119 | **117** | 128 | 127 | 133 | **129** | 122 | 132 | 130 | **128** | 111 | 122 | 119 | **117** |  |  |  |  |
|  | RSD (%) | 12.3 | 5.1 | 12.7 | **10.9** | 9.4 | 14.5 | 6.2 | **9.5** | 4.8 | 7.4 | 5.1 | **6.5** | 4.7 | 8.8 | 3.1 | **7.0** |  |  |  |  |
| 14 | **BPS** |  |  |  |  |  |  |  |  |  |  |  |  |  |  |  |  |  |  |  |  |
|  | AM (ng mL^-1^) | 0.883 | 0.926 | 0.804 | **0.871** | 3.73 | 3.89 | 3.75 | **3.79** | 11.4 | 12.1 | 11.1 | **11.5** | 29.2 | 31.0 | 30.2 | **30.1** |  |  |  |  |
|  | Accuracy (%) | 88.3 | 92.6 | 80.4 | **87.1** | 93.2 | 97.1 | 93.7 | **94.7** | 95.1 | 101 | 92.2 | **95.9** | 97.2 | 103 | 101 | **100** |  |  |  |  |
|  | RSD (%) | 4.4 | 9.9 | 6.1 | **9.1** | 5.5 | 3.0 | 8.3 | **5.9** | 7.0 | 6.7 | 4.6 | **7.0** | 7.0 | 8.4 | 6.3 | **7.3** |  |  |  |  |
| 15 | **BP-1** |  |  |  |  |  |  |  |  |  |  |  |  |  |  |  |  |  |  |  |  |
|  | AM (ng mL^-1^) | 1.07 | 1.03 | 1.06 | **1.05** | 3.94 | 3.84 | 4.14 | **3.96** | 11.4 | 11.7 | 11.8 | **11.6** | 28.7 | 30.5 | 30.9 | **30.0** | 202 | 213 | 208 | **208** |
|  | Accuracy (%) | 107 | 103 | 106 | **105** | 98.5 | 96.0 | 86.2 | **93.6** | 94.7 | 97.2 | 98.1 | **96.7** | 95.6 | 102 | 103 | **100** | 101 | 107 | 104 | **104** |
|  | RSD (%) | 12.6 | 11.2 | 7.5 | **10.2** | 6.4 | 7.7 | 7.5 | **7.4** | 5.0 | 8.7 | 4.8 | **6.2** | 3.7 | 10.3 | 6.9 | **7.8** | 7.5 | 7.9 | 6.5 | **7.2** |
| 16 | **BP-3** |  |  |  |  |  |  |  |  |  |  |  |  |  |  |  |  |  |  |  |  |
|  | AM (ng mL^-1^) | 0.970 | 0.787 | 0.781 | **0.854** | 3.99 | 3.74 | 4.08 | **3.94** | 12.3 | 11.1 | 11.3 | **11.6** | 30.4 | 27.9 | 29.2 | **29.2** | 220 | 203 | 200 | **208** |
|  | Accuracy (%) | 97.0 | 78.7 | 73.2. | **85.4** | 99.7 | 93.5 | 102 | **98.4** | 103 | 92.7 | 94.3 | **96.6** | 101 | 93.0 | 97.3 | **97.2** | 110 | 101 | 100 | **104** |
|  | RSD (%) | 7.7 | 19.9 | 22.8 | **17.1** | 5.4 | 7.9 | 5.1 | **6.9** | 9.7 | 7.6 | 9.5 | **9.7** | 6.6 | 8.6 | 10.5 | **8.9** | 8.1 | 5.2 | 6.1 | **7.6** |
| 17 | **TCS** |  |  |  |  |  |  |  |  |  |  |  |  |  |  |  |  |  |  |  |  |
|  | AM (ng mL^-1^) | 0.975 | 1.05 | 1.09 | **1.04** | 4.34 | 4.45 | 4.33 | **4.37** | 12.5 | 12.9 | 12.3 | **12.6** | 29.8 | 30.3 | 30.6 | **30.2** | 179 | 199 | 180 | **186** |
|  | Accuracy (%) | 97.5 | 105 | 109 | **104** | 109 | 111 | 108 | **109** | 104 | 108 | 102 | **105** | 99.4 | 101 | 102 | **101** | 89.4 | 99.4 | 90.1 | **93.2** |
|  | RSD (%) | 11.9 | 8.9 | 11.1 | **10.7** | 5.3 | 8.5 | 13.6 | **9.2** | 2.3 | 8.8 | 4.5 | **6.1** | 4.4 | 6.2 | 4.8 | **5.0** | 8.0 | 5.4 | 8.9 | **8.6** |
| 18 | **2,4-DCP** |  |  |  |  |  |  |  |  |  |  |  |  |  |  |  |  |  |  |  |  |
|  | AM (ng mL^-1^) | 1.06 | 0.937 | 0.878 | **0.959** | 4.28 | 3.62 | 3.49 | **3.80** | 12.7 | 10.7 | 9.74 | **11.0** |  |  |  |  |  |  |  |  |
|  | Accuracy (%) | 106 | 93.7 | 87.8 | **95.9** | 107 | 90.4 | 87.2 | **94.9** | 106 | 89.0 | 81.2 | **92.1** |  |  |  |  |  |  |  |  |
|  | RSD (%) | 7.8 | 17.1 | 16.2 | **15.2** | 6.5 | 14.7 | 12.3 | **14.1** | 5.1 | 12.0 | 10.0 | **14.4** |  |  |  |  |  |  |  |  |
| 19 | **2,5-DCP** |  |  |  |  |  |  |  |  |  |  |  |  |  |  |  |  |  |  |  |  |
|  | AM (ng mL^-1^) | 0.968 | 0.993 | 0.969 | **0.976** | 3.96 | 4.12 | 4.17 | **4.08** | 11.8 | 11.8 | 11.1 | **11.6** | 31.1 | 34.0 | 32.7 | **32.6** |  |  |  |  |
|  | Accuracy (%) | 96.8 | 99.3 | 96.9 | **97.6** | 99.1 | 103 | 104 | **102** | 98.5 | 98.2 | 92.4 | **96.3** | 104 | 113 | 109 | **109** |  |  |  |  |
|  | RSD (%) | 6.6 | 10.4 | 13.4 | **10.0** | 3.2 | 9.3 | 9.5 | **7.8** | 5.7 | 7.0 | 5.8 | **6.6** | 4.0 | 9.5 | 7.8 | **8.1** |  |  |  |  |
| 20 | **PCP** |  |  |  |  |  |  |  |  |  |  |  |  |  |  |  |  |  |  |  |  |
|  | AM (ng mL^-1^) | 0.543 | 0.608 | 0.532 | **0.563** | 2.57 | 2.96 | 2.38 | **2.64** | 8.59 | 9.60 | 7.66 | **8.62** | 22.8 | 27.6 | 24.6 | **25.3** |  |  |  |  |
|  | Accuracy (%) | 54.3 | 60.8 | 53.2 | **56.3** | 64.3 | 74.0 | 59.4 | **66.1** | 71.6 | 80.0 | 63.8 | **71.8** | 76.1 | 92.1 | 82.0 | **84.3** |  |  |  |  |
|  | RSD (%) | 16.2 | 34.9 | 28.0 | **28.2** | 27.1 | 16.3 | 37.0 | **26.8** | 23.0 | 4.0 | 14.8 | **16.5** | 19.1 | 16.8 | 3.7 | **15.4** |  |  |  |  |
| 21 | **TCPy**r |  |  |  |  |  |  |  |  |  |  |  |  |  |  |  |  |  |  |  |  |
|  | AM (ng mL^-1^) | 1.08 | 1.14 | 1.16 | **1.12** | 4.36 | 4.36 | 4.44 | **4.39** | 12.8 | 12.7 | 11.8 | **12.4** | 30.3 | 30.4 | 31.4 | **30.7** |  |  |  |  |
|  | Accuracy (%) | 108 | 114 | 116 | **112** | 109 | 109 | 111 | **110** | 107 | 106 | 98.7 | **104** | 101 | 101 | 105 | **102** |  |  |  |  |
|  | RSD (%) | 7.5 | 11.5 | 10.8 | **11.5** | 3.5 | 6.9 | 7.2 | **5.8** | 2.7 | 5.2 | 5.1 | **5.6** | 3.5 | 5.6 | 5.7 | **5.0** |  |  |  |  |
| 22 | **6-CNA** |  |  |  |  |  |  |  |  |  |  |  |  |  |  |  |  |  |  |  |  |
|  | AM (ng mL^-1^) | 0.801 | 0.638 | 0.657 | **0.698** | 3.61 | 3.21 | 3.50 | **3.44** | 11.4 | 9.80 | 9.71 | **10.3** | 31.9 | 26.8 | 30.1 | **29.6** |  |  |  |  |
|  | Accuracy (%) | 80.1 | 63.8 | 65.7 | **69.8** | 90.3 | 80.1 | 87.6 | **86.0** | 94.7 | 81.6 | 80.9 | **85.7** | 106 | 89.3 | 100 | **98.7** |  |  |  |  |
|  | RSD (%) | 12.5 | 14.9 | 24.9 | **19.8** | 13.2 | 17.7 | 13.8 | **14.9** | 12.8 | 10.7 | 8.5 | **12.9** | 12.8 | 8.7 | 12.9 | **13.4** |  |  |  |  |
| 23 | **1-NP** |  |  |  |  |  |  |  |  |  |  |  |  |  |  |  |  |  |  |  |  |
|  | AM (ng mL^-1^) | 0.940 | 0.925 | 0.990 | **0.951** | 3.85 | 3.97 | 4.16 | **3.99** | 11.6 | 11.9 | 12.0 | **11.8** | 29.8 | 30.9 | 32.2 | **30.9** |  |  |  |  |
|  | Accuracy (%) | 94.0 | 92.5 | 99.0 | **95.1** | 96.3 | 99.2 | 104 | **99.8** | 96.7 | 99.0 | 100 | **98.6** | 99.3 | 103 | 107 | **103** |  |  |  |  |
|  | RSD (%) | 8.9 | 7.7 | 7.8 | **8.2** | 6.6 | 3.2 | 6.8 | **6.3** | 8.1 | 7.0 | 7.5 | **7.2** | 8.0 | 8.5 | 7.0 | **8.0** |  |  |  |  |
| 24 | **2-NP** |  |  |  |  |  |  |  |  |  |  |  |  |  |  |  |  |  |  |  |  |
|  | AM (ng mL^-1^) | 0.837 | 0.780 | 0.781 | **0.799** | 4.39 | 3.81 | 3.94 | **4.04** | 11.9 | 10.8 | 10.6 | **11.1** | 28.8 | 26.1 | 26.1 | **27.0** | 187 | 170 | 162 | **173** |
|  | Accuracy (%) | 83.7 | 78.0 | 78.1 | **79.9** | 110 | 95.3 | 98.4 | **101** | 98.9 | 90.1 | 88.0 | **92.3** | 96.2 | 86.9 | 87.0 | **90.0** | 93.4 | 84.9 | 81.0 | **86.5** |
|  | RSD (%) | 9.5 | 9.9 | 8.7 | **9.4** | 3.5 | 8.0 | 4.9 | **8.2** | 8.5 | 4.2 | 4.9 | **8.0** | 4.0 | 6.3 | 6.9 | **7.4** | 8.4 | 8.0 | 4.5 | **9.3** |
| 25 | **3-PBA** |  |  |  |  |  |  |  |  |  |  |  |  |  |  |  |  |  |  |  |  |
|  | AM (ng mL^-1^) | 0.919 | 0.883 | 0.927 | **0.910** | 3.95 | 3.84 | 3.97 | **3.92** | 12.2 | 12.3 | 12.0 | **12.2** | 31.7 | 32.1 | 32.3 | **32.0** |  |  |  |  |
|  | Accuracy (%) | 91.9 | 88.3 | 92.7 | **91.0** | 98.6 | 96.0 | 99.1 | **97.9** | 102 | 103 | 100 | **101** | 106 | 107 | 108 | **107** |  |  |  |  |
|  | RSD (%) | 7.3 | 3.9 | 12.5 | **8.4** | 5.0 | 7.1 | 5.6 | **5.8** | 5.1 | 3.6 | 3.7 | **4.1** | 3.5 | 8.1 | 3.8 | **5.3** |  |  |  |  |
| 26 | **4OH3PBA** |  |  |  |  |  |  |  |  |  |  |  |  |  |  |  |  |  |  |  |  |
|  | AM (ng mL^-1^) | 0.877 | 1.06 | 1.02 | **0.983** | 3.69 | 3.90 | 3.82 | **3.81** | 10.8 | 12.3 | 11.3 | **11.5** | 29.6 | 35.3 | 30.8 | **31.8** |  |  |  |  |
|  | Accuracy (%) | 87.7 | 106 | 102 | **98.3** | 92.3 | 97.5 | 95.4 | **95.2** | 89.7 | 103 | 93.9 | **95.8** | 98.7 | 118 | 103 | **106** |  |  |  |  |
|  | RSD (%) | 16.9 | 9.0 | 6.1 | **13.3** | 13.3 | 8.9 | 5.2 | **9.0** | 14.6 | 12.8 | 11.3 | **13.3** | 7.2 | 7.1 | 6.8 | **10.2** |  |  |  |  |

AM – arithmetic mean; RSD – relative standard deviation

**Table S4** Comparison of urinary concentration of 27 biomarkers in 24-h urine samples between different studies

|  | Poland | | Poland | | Germany | | Germany | | Norway | | Netherlands | |
| --- | --- | --- | --- | --- | --- | --- | --- | --- | --- | --- | --- | --- |
|  | This study | | (Wielgomas, 2013) | | (Koch et al., 2012) | | (Moos et al., 2015) | | (Husøy et al., 2019) | | (van der Meer et al., 2020) | |
|  | DF  (%) | Median  (ng mL^-1^) | DF  (%) | Median  (ng mL^-1^) | DF  (%) | Median  (ng mL^-1^) | DF  (%) | Median  (ng mL^-1^) | DF  (%) | Median  (ng mL^-1^) | DF  (%) | Median  (ng mL^-1^) |
| MeP | 97.4 | 18.9 |  |  |  |  | 99 | 39.8 | 100 | 7.45 | 100 | 26.85 |
| EtP | 86.8 | 3.72 |  |  |  |  | 79 | 2.1 | 99 | 1.13 | 98 | 1.68 |
| PrP | 78.9 | 1.01 |  |  |  |  | 81 | 4.8 | 65 | 0.28 | 93 | 2.70 |
| iBuP | 28.9 | nc |  |  |  |  | 24 | <LOQ |  |  |  |  |
| BuP | 81.6 | 0.761 |  |  |  |  | 40 | <LOQ | 50 | 0.13 | 86 | 0.16 |
| BPA | 94.7 | 1.35 |  |  | 99.8 | 1.55 |  |  | 96 | 1.36 | 95 | 1.9 |
| BPAF | 5.3 | nc |  |  |  |  |  |  |  |  |  |  |
| BPB | 0.0 | nc |  |  |  |  |  |  |  |  |  |  |
| BPBP | 0.0 | nc |  |  |  |  |  |  |  |  |  |  |
| BPC | 0.0 | nc |  |  |  |  |  |  |  |  |  |  |
| BPE | 0.0 | nc |  |  |  |  |  |  |  |  |  |  |
| BPF | 28.9 | nc |  |  |  |  |  |  | 4 | 0.08 | 52 | 0.24 |
| BPG | 0.0 | nc |  |  |  |  |  |  |  |  |  |  |
| BPS | 21.1 | nc |  |  |  |  |  |  | 29 | 0.16 | 9 | <LOD |
| BP-1 | 36.8 | nc |  |  |  |  |  |  |  |  |  |  |
| BP-3 | 57.9 | 0.380 |  |  |  |  |  |  | 100 | 3.72 |  |  |
| TCS | 50.0 | 0.311 |  |  |  |  |  |  | 93 | 0.19 |  |  |
| 24-DCP | 44.7 | nc |  |  |  |  |  |  |  |  |  |  |
| 2,5-DCP | 65.8 | 0.448 |  |  |  |  |  |  |  |  |  |  |
| PCP | 2.6 | nc |  |  |  |  |  |  |  |  |  |  |
| TCPyr | 94.7 | 2.07 |  |  |  |  |  |  |  |  |  |  |
| 6-CNA | 0.0 | nc |  |  |  |  |  |  |  |  |  |  |
| 1-NP | 44.7 | nc |  |  |  |  |  |  |  |  |  |  |
| 2-NP | 100.0 | 2.38 |  |  |  |  |  |  |  |  |  |  |
| 3-PBA | 23.7 | nc | 93.7 | 0.270 |  |  |  |  |  |  |  |  |
| 4OH3PBA | 7.9 | nc |  |  |  |  |  |  |  |  |  |  |

DF – detection frequency; nc- not calculated (detection frequency <50%)

**Table S5** Comparison of analytical methods used for quantification of urinary biomarkers

| Analytes | Sample volume (mL) | Extraction | Instrumental technique | LOD (ng mL^-1^) | Reference |
| --- | --- | --- | --- | --- | --- |
| Total: 12 analytes  4 parabens, BP-3, 7 phthalate metabolites | 3.0 | SPE (Bond Elut Certify LRC, 130 mg, 10 mL) | UPLC/MS/MS | 0.09-0.37 | (Dewalque et al., 2014) |
| Total: 5 analytes  5 parabens | 0.5 | SPE (Strata XL; 200 mg, 3 mL) | LC/MS/MS | 0.07-0.40 | (Frederiksen et al., 2011) |
| Total: 121 analytes  45 plasticizers and metabolites, 34 environmental phenols, 31 pesticides, 11 OH-PAHs | 0.5 | SPE (ABS Elut NEXUS, 60 mg, 3 mL) | LC/MS/MS (two injections) | 0.01-5.0 | (Zhu et al., 2021) |
| Total: 9 analytes  BPA, BP-3, TCS, 6 environmental phenols | 0.1 | on-line SPE (TurboFlow Cyclone-P, 0.5 mm × 50 mm) | LC/MS/MS | 0.01-0.13 | (Frederiksen et al., 2013) |
| Total: 16 analytes  9 parabens, BPA, 3 benzophenones, 3 environmental phenols | 0.1 | on-line SPE (LiChrospher RP-8 ADS, 25 µm) | LC/MS/MS | 0.5-2.0 (LOQ) | (Moos et al., 2014) |
| Total: 5 analytes  5 parabens | 0.1 | on-line SPE (LiChrosphere  RP-18 ADS, 25 µm) | LC/MS/MS | 0.1-0.18 | (Ye et al., 2006) |
| Total: 20 analytes  5 parabens, BPA, BP-3, TCS, 12 environmental phenols | 0.5 | LLE (MTBE:hexane (1:3, *v:v*),  SPE (K_2_CO_3_-treated silica gel) | GC/MS/MS | 0.7-9.8 pg/mL | (Lu et al., 2015) |
| Total: 3 analytes  BPA, TCS, 4-nonylphenol | 3.0 | SPE (OASIS HLB, 60 mg, 3 ml) | GC/MS/MS | 0.16-0.33 | (Pirard et al., 2012) |
| Total: 22 analytes  5 parabens, 6 bisphenols, 2 benzophenones, TCS, 4 phenolic metabolites of pesticides, 4 acidic metabolites of pesticides | 2.0 | SPE (Bond Elut Plexa (30 mg, 1 mL) | GC/MS/MS | 0.1-0.5 | This study |

**References:**

Dewalque, L., Pirard, C., Dubois, N., Charlier, C., 2014. Simultaneous determination of some phthalate metabolites, parabens and benzophenone-3 in urine by ultra high pressure liquid chromatography tandem mass spectrometry. J Chromatogr B Analyt Technol Biomed Life Sci 949–950, 37–47. https://doi.org/10.1016/j.jchromb.2014.01.002

Frederiksen, H., Aksglaede, L., Sorensen, K., Nielsen, O., Main, K.M., Skakkebaek, N.E., Juul, A., Andersson, A.M., 2013. Bisphenol A and other phenols in urine from Danish children and adolescents analyzed by isotope diluted TurboFlow-LC-MS/MS. Int J Hyg Environ Health 216, 710–720. https://doi.org/10.1016/j.ijheh.2013.01.007

Frederiksen, H., Jørgensen, N., Andersson, A.-M., 2011. Parabens in urine, serum and seminal plasma from healthy Danish men determined by liquid chromatography-tandem mass spectrometry (LC-MS/MS). J Expo Sci Environ Epidemiol 21, 262–271. https://doi.org/10.1038/jes.2010.6

Husøy, T., Andreassen, M., Hjertholm, H., Carlsen, M.H., Norberg, N., Sprong, C., Papadopoulou, E., Sakhi, A.K., Sabaredzovic, A., Dirven, H.A.A.M., 2019. The Norwegian biomonitoring study from the EU project EuroMix: Levels of phenols and phthalates in 24-hour urine samples and exposure sources from food and personal care products. Environ Int 132, 105103. https://doi.org/10.1016/j.envint.2019.105103

Koch, H.M., Kolossa-Gehring, M., Schröter-Kermani, C., Angerer, J., Brüning, T., 2012. Bisphenol A in 24 h urine and plasma samples of the German Environmental Specimen Bank from 1995 to 2009: A retrospective exposure evaluation. J Expo Sci Environ Epidemiol 22, 610–616. https://doi.org/10.1038/jes.2012.39

Lu, D., Feng, C., Wang, D., Lin, Y., Ip, H.S.S., She, J., Xu, Q., Wu, C., Wang, G., Zhou, Z., 2015. Analysis of twenty phenolic compounds in human urine: hydrochloric acid hydrolysis, solid-phase extraction based on K2CO 3-treated silica, and gas chromatography tandem mass spectrometry. Anal Bioanal Chem 407, 4131–41. https://doi.org/10.1007/s00216-015-8598-1

Moos, R.K., Angerer, J., Wittsiepe, J., Wilhelm, M., Brüning, T., Koch, H.M., 2014. Rapid determination of nine parabens and seven other environmental phenols in urine samples of German children and adults. Int J Hyg Environ Health 217, 845–853. https://doi.org/10.1016/j.ijheh.2014.06.003

Moos, R.K., Koch, H.M., Angerer, J., Apel, P., Schröter-Kermani, C., Brüning, T., Kolossa-Gehring, M., 2015. Parabens in 24h urine samples of the German Environmental Specimen Bank from 1995 to 2012. Int J Hyg Environ Health 218, 666–674. https://doi.org/10.1016/j.ijheh.2015.07.005

Pirard, C., Sagot, C., Deville, M., Dubois, N., Charlier, C., 2012. Urinary levels of bisphenol A, triclosan and 4-nonylphenol in a general Belgian population. Environ Int 48, 78–83. https://doi.org/10.1016/j.envint.2012.07.003

van der Meer, T.P., van Faassen, M., van Beek, A.P., Snieder, H., Kema, I.P., Wolffenbuttel, B.H.R., van Vliet-Ostaptchouk, J. v., 2020. Exposure to Endocrine Disrupting Chemicals in the Dutch general population is associated with adiposity-related traits. Sci Rep 10, 1–10. https://doi.org/10.1038/s41598-020-66284-3

Wielgomas, B., 2013. Variability of urinary excretion of pyrethroid metabolites in seven persons over seven consecutive days—Implications for observational studies. Toxicol Lett 221, 15–22. https://doi.org/10.1016/j.toxlet.2013.05.009

Ye, X., Kuklenyik, Z., Bishop, A.M., Needham, L.L., Calafat, A.M., 2006. Quantification of the urinary concentrations of parabens in humans by on-line solid phase extraction-high performance liquid chromatography-isotope dilution tandem mass spectrometry. J Chromatogr B Analyt Technol Biomed Life Sci 844, 53–9. https://doi.org/10.1016/j.jchromb.2006.06.037

Zhu, H., Chinthakindi, S., Kannan, K., 2021. A method for the analysis of 121 multi-class environmental chemicals in urine by high-performance liquid chromatography-tandem mass spectrometry. J Chromatogr A 1646, 462146. https://doi.org/10.1016/J.CHROMA.2021.462146


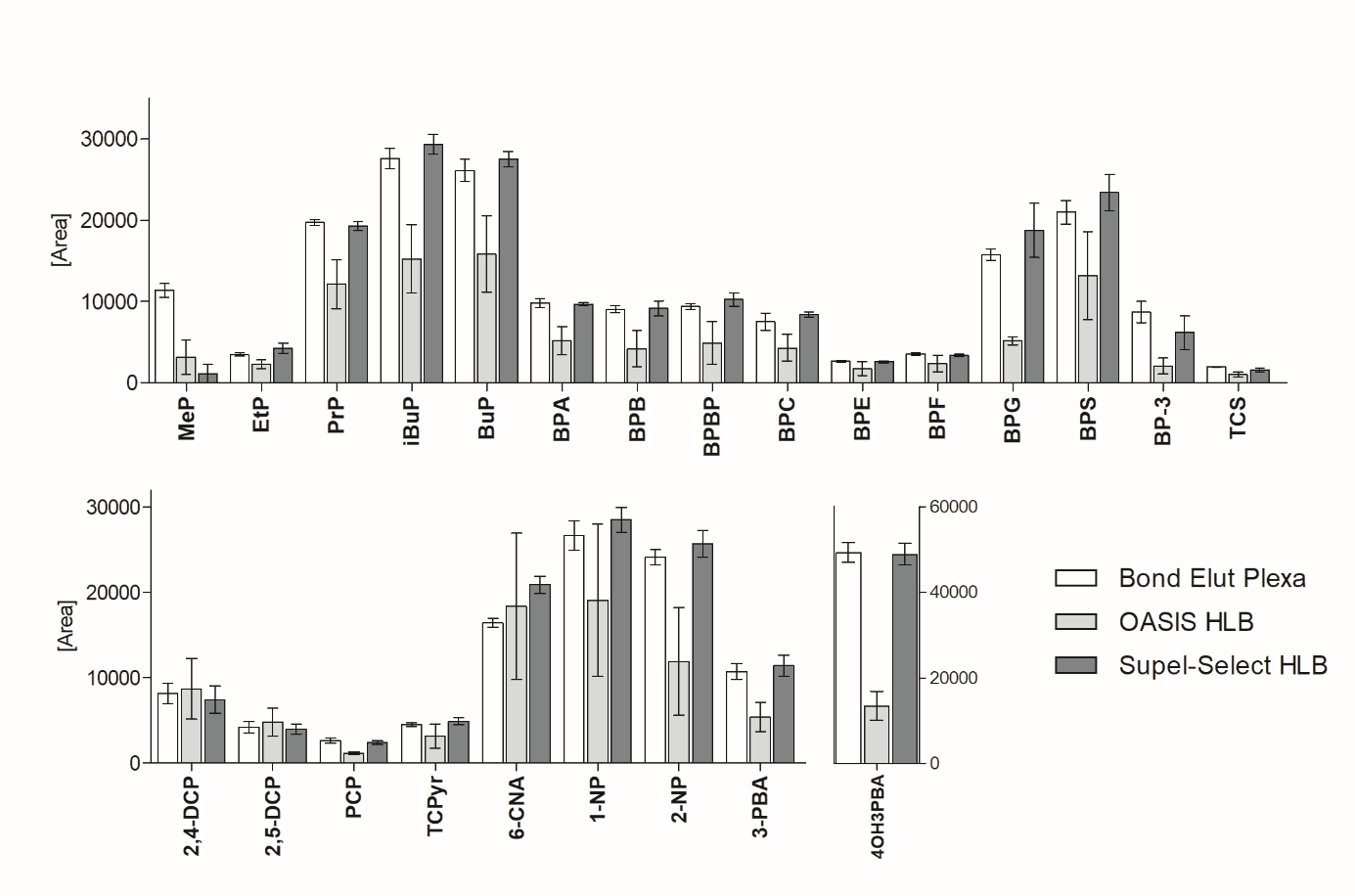


**Fig. S1** Comparison of the extraction efficiency of all biomarkers from urine samples using three SPE sorbents


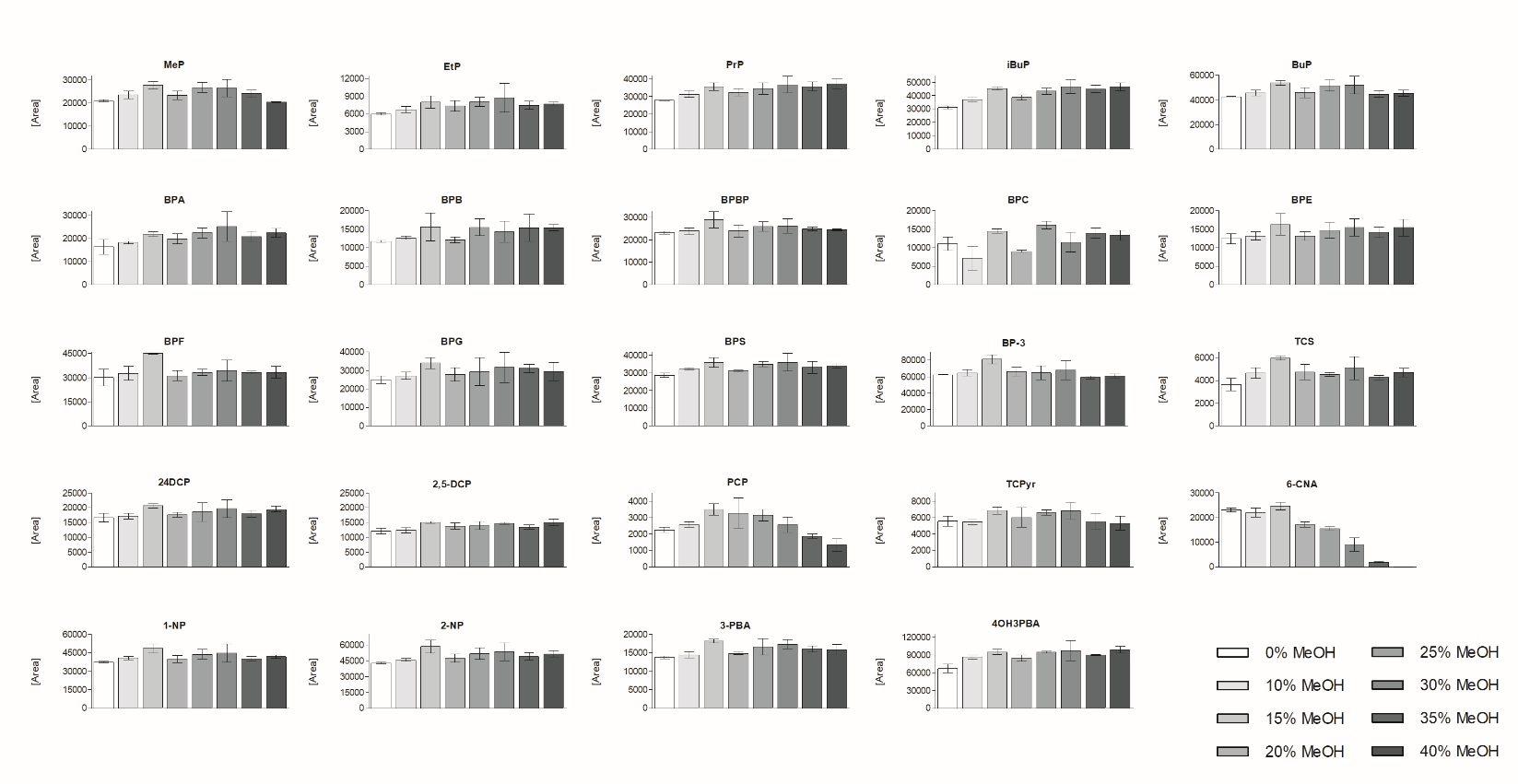


**Fig. S2** Evaluation of the sorbent washing step. All washing solvents were prepared in 1% HCOOH in water.


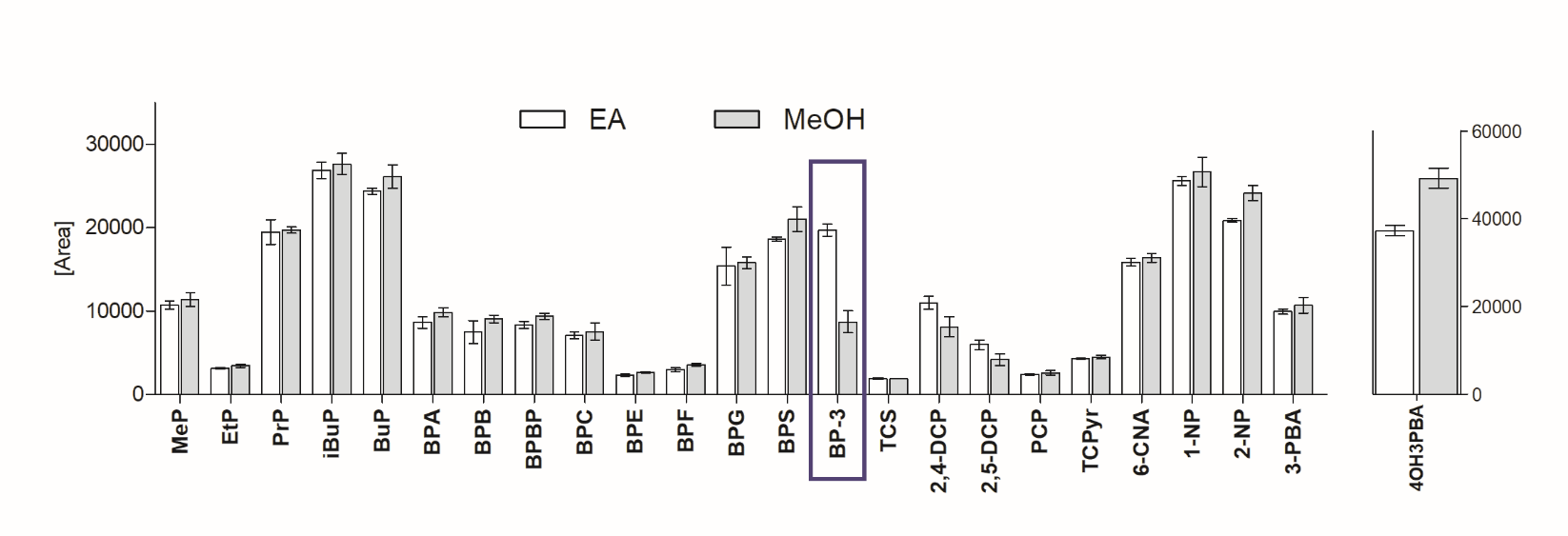


**Fig. S3** Comparison of ethyl acetate (EA) and methanol (MeOH) as an elution solvent


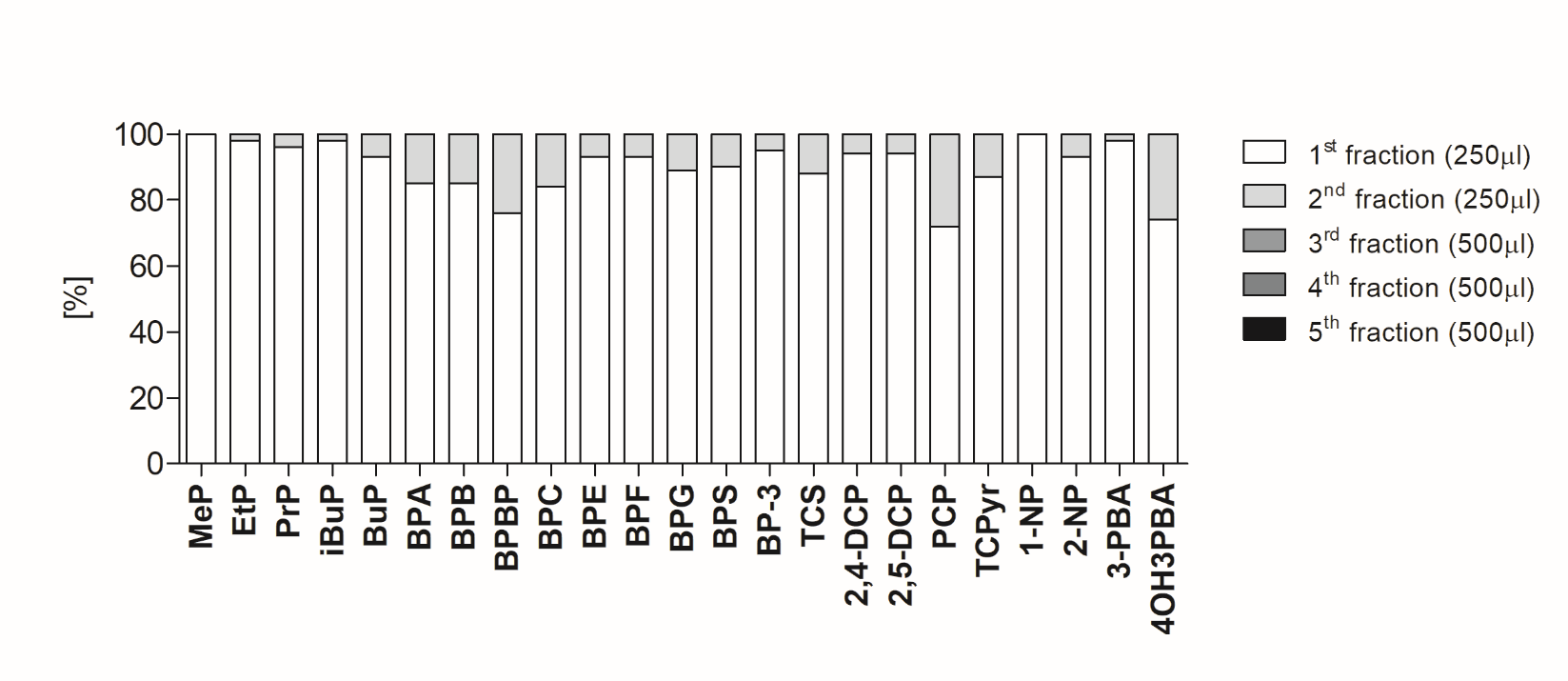


**Fig. S4** Elution profile of all analytes generated using ethyl acetate as an elution solvent


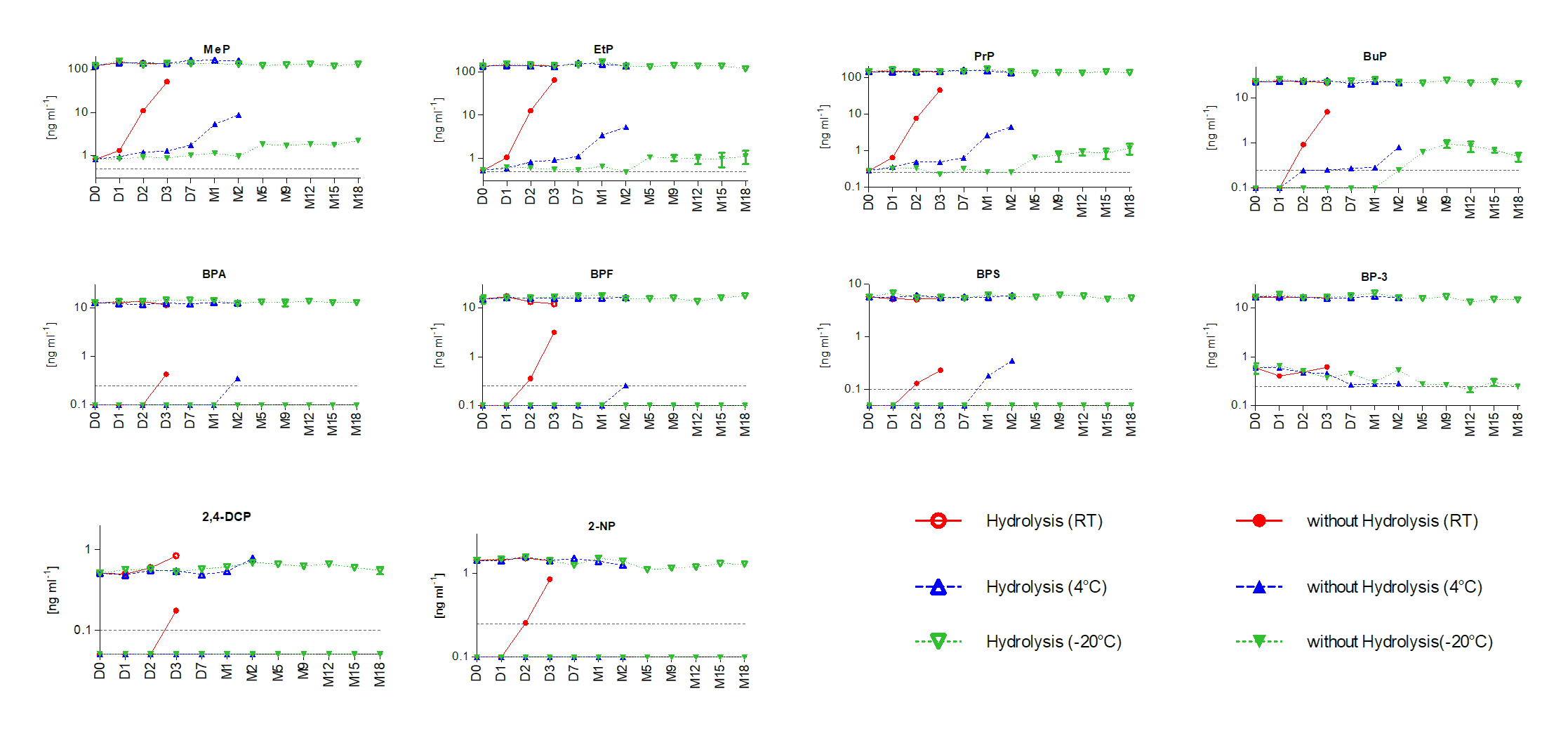


**Fig. S5** Stability of conjugate concentrations in the urine presented for selected biomarkers. The samples were stored at three different temperatures: room temperature (RT), 4°C, and -20°C. Before SPE, samples were enzymatically treated (Hydrolysis) or analyzed without this step (without Hydrolysis). The grey, dotted line represents LOQ


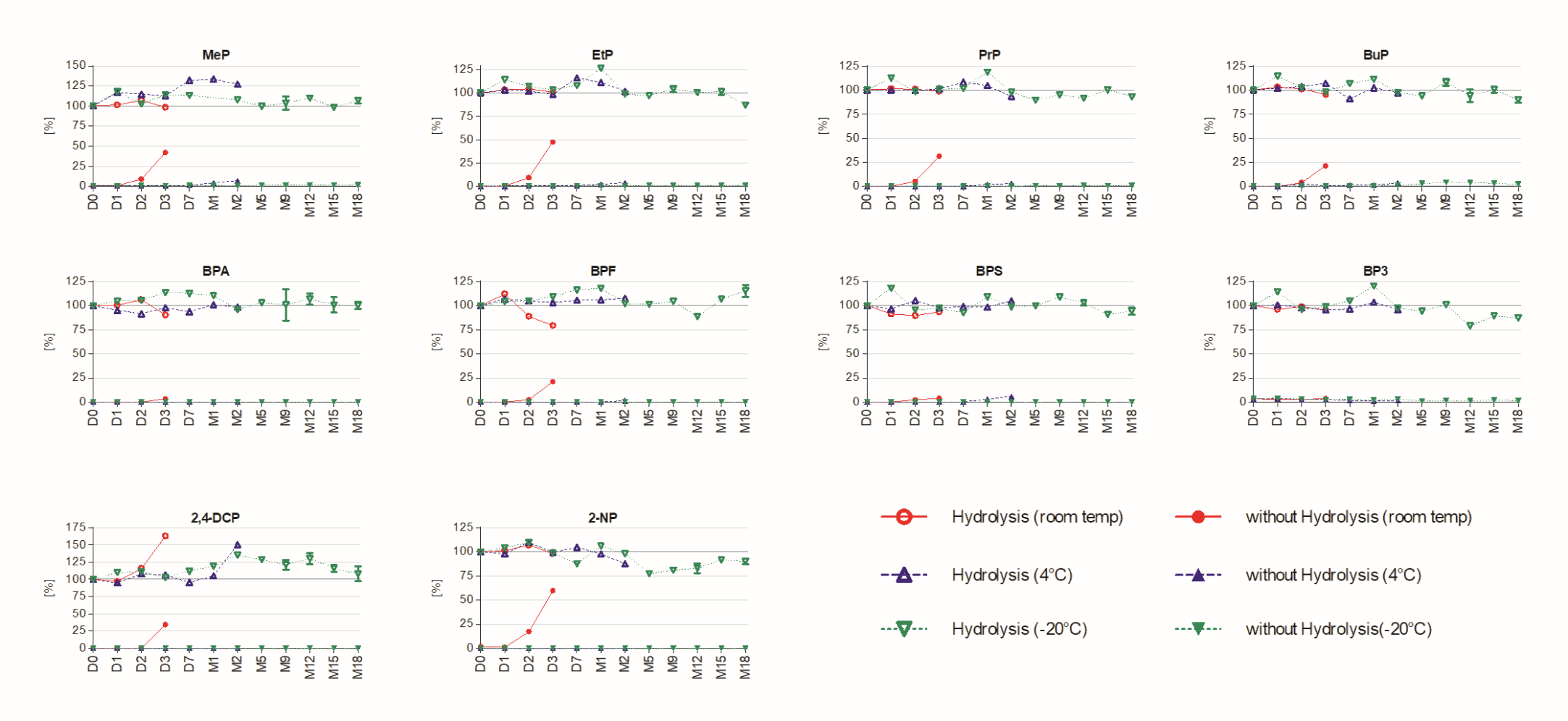


**Fig. S6** Stability of biomarker conjugates in the urine presented as a normalized response (100% represents the concentration of biomarkers on D0). The samples were stored at three different temperatures: room temperature (RT), 4°C, and -20°C. Before SPE, samples were enzymatically treated (Hydrolysis) or analyzed without this step (without Hydrolysis)
